# Genetic risk, adherence to a healthy lifestyle, and type 2 diabetes risk among 550,000 Chinese adults: results from 2 independent Asian cohorts

**DOI:** 10.1093/ajcn/nqz310

**Published:** 2020-01-24

**Authors:** Haoxin Li, Chiea-Chuen Khor, Junning Fan, Jun Lv, Canqing Yu, Yu Guo, Zheng Bian, Ling Yang, Iona Y Millwood, Robin G Walters, Yiping Chen, Jian-Min Yuan, Yan Yang, Chen Hu, Junshi Chen, Zhengming Chen, Woon-Puay Koh, Tao Huang, Liming Li

**Affiliations:** 1 Department of Epidemiology and Biostatistics, School of Public Health, Peking University Health Science Center, Beijing, China; 2 Genome Institute of Singapore, Singapore; 3 Singapore Eye Research Institute, Singapore; 4 Key Laboratory of Molecular Cardiovascular Sciences (Peking University), Ministry of Education, Beijing, China; 5 Peking University Institute of Environmental Medicine, Beijing, China; 6 Chinese Academy of Medical Sciences, Beijing, China; 7 Clinical Trial Service Unit & Epidemiological Studies Unit, Nuffield Department of Population Health, University of Oxford, Oxford, United Kingdom; 8 Division of Cancer Control and Population Sciences, University of Pittsburgh Cancer Institute, Pittsburgh, PA, USA; 9 Department of Epidemiology, University of Pittsburgh Graduate School of Public Health, Pittsburgh, PA, USA; 10 Huixian People's Hospital, Huixian, Henan, China; 11 NCDs Prevention and Control Department, Huixian CDC, Huixian, Henan, China; 12 China National Center for Food Safety Risk Assessment, Beijing, China; 13 Health Services and Systems Research, Duke-NUS Medical School, Singapore; 14 Saw Swee Hock School of Public Health, National University of Singapore, Singapore

**Keywords:** type 2 diabetes, genetics, gene–environment interaction, Chinese, lifestyle

## Abstract

**Background:**

Whether genetic susceptibility to type 2 diabetes is modified by a healthy lifestyle among Chinese remains unknown.

**Objectives:**

The aim of the study was to determine whether genetic risk and adherence to a healthy lifestyle contribute independently to the risk of developing type 2 diabetes.

**Methods:**

We defined a lifestyle score using BMI, alcohol intake, smoking, physical activities, and diets in 461,030 participants from the China Kadoorie Biobank and 38,434 participants from the Singapore Chinese Health Study. A genetic risk score was constructed based on type 2 diabetes loci among 100,175 and 16,172 participants in each cohort, respectively. A Cox proportional-hazards model was used to estimate the interaction between genetic and lifestyle factors on the risk of type 2 diabetes.

**Results:**

In 2 independent Asian cohorts, we consistently found a healthy lifestyle (the bottom quintile of lifestyle score) was associated with a substantially lower risk of type 2 diabetes than an unhealthy lifestyle (the top quintile of lifestyle score) regardless of genetic risk. In those at a high genetic risk, the risk of type 2 diabetes was 57% lower among participants with a healthy lifestyle than among those with an unhealthy lifestyle in the pooled cohorts. Among participants at high genetic risk, the standardized 10-y incidence of type 2 diabetes was 7.11% in those with an unhealthy lifestyle vs. 2.45% in those with a healthy lifestyle.

**Conclusions:**

In 2 independent cohorts involving 558,302 Chinese participants, we did not observe an interaction between genetics and lifestyle with type 2 diabetes risk, but our findings provide replicable evidence to show lifestyle factors and genetic factors were independently associated with the risk of type 2 diabetes. Within any genetic risk category, a healthy lifestyle was associated with a significantly lower risk of type 2 diabetes among the Chinese population.

## Introduction

Over the past several decades, the diabetes burden has increased rapidly around the world (1). The number of diabetes cases continues to increase in China, which has the largest diabetic population in the world (2–4). It is well-known that type 2 diabetes is caused by both genetic and lifestyle factors (5, 6). To date, genome-wide association studies (GWASs) have identified >100 independent loci for type 2 diabetes (7). It has been documented that genetic risk score (GRS), calculated based on the identified risk alleles, is predictive of incident type 2 diabetes and provides a continuous and quantitative measure of genetic susceptibility (8).

Compelling observational studies have also shown that healthy lifestyle factors such as lower BMI (9), moderate drinking (10), no smoking (11), balanced dietary pattern (12), and more physical activities (13) were associated with lower risk of type 2 diabetes. We previously reported that Chinese adults with >5 healthy lifestyle factors were at 83% lower risk of type 2 diabetes than those without healthy lifestyle factors (14). Importantly, evidence from Europeans has suggested that lifestyle factors such as diet, nutrients, and BMI modulate the genetic susceptibility to the risk of type 2 diabetes (15–17). For example, a stronger genetic association was observed among obese participants in the United States (17) and among participants with a Western dietary pattern in Europe (15). However, whether genetic susceptibility to type 2 diabetes is attenuated by a healthy lifestyle remains unknown among Chinese adults.

To help fill these gaps, we analyzed baseline data among 550,000 participants in 2 independent Asian prospective cohorts: the China Kadoorie Biobank (CKB) and the Singapore Chinese Health Study (SCHS). The aims of our study were *1*) to test the hypothesis that both genetic factors and lifestyle factors contributed independently to the risk of developing type 2 diabetes; *2*) to determine the extent to which healthy lifestyle factors are associated with a lower risk of type 2 diabetes among participants with a high genetic risk; and *3*) to estimate the joint associations of genetic factors and lifestyle factors with the risk of type 2 diabetes.

## Methods

### Discovery cohort

The CKB is a prospective cohort that included 512,891 participants aged 30–79 y from 10 study areas including 5 urban areas and 5 rural areas in China. The baseline data were collected by validated questionnaire and physical measurements. Written informed consent forms from all participants were obtained between 2004 and 2008. Details of the CKB cohort and characteristics of the study participants have been described in a previous publication (18). The Ethical Review Committee of the Chinese Center for Disease Control and Prevention (Beijing, China) and the Oxford Tropical Research Ethics Committee, University of Oxford (Oxford, United Kingdom) approved the study.

### Replication cohort

The SCHS was established between April 1993 and December 1998 when investigators recruited 35,303 Chinese women and 27,954 Chinese men aged 45–74 y and living in Singapore. All participants were interviewed in person by structured questionnaires and surviving participants received a follow-up via telephone call at follow-up I (1999–2004) and follow-up II (2006–2010). Details of the SCHS cohort have been described elsewhere (19). The study was approved by the institutional review board at the National University of Singapore and all participants gave informed consent.

### Lifestyle and covariates

In the CKB cohort, diet and lifestyle factors were assessed by trained staff with baseline questionnaires. For alcohol information, we acquired typical drinking frequency, types of alcoholic beverage drunk usually, and volume of drinking alcohol on a typical drinking day in the past 12 mo. Smoking questions included smoking status and frequency, and the amounts and types of tobacco smoked per day for ever smokers. A short qualitative FFQ was used to assess habitual intakes of 12 conventional food groups in the past 12 mo. We asked about the usual types and duration of activities in occupational, domestic, and leisure-time-related domains and commuting in the past 12 mo. We multiplied the metabolic equivalent tasks (METs) value for a particular type of activity by hours spent on that activity per day and summed the MET-hours for all activities to acquire the daily amount of physical activity. Weight, height, and waist and hip circumferences were measured by trained staff with calibrated instruments. BMI was calculated as kg/m^2^. Waist-to-hip ratio (WHR) was the ratio of waist circumference to hip circumference. If a participant had ≥1 first-degree relative suffering from diabetes, he or she was considered as having a family history of diabetes. We have previously validated the reproducibility of the assessment (20–22).

In the SCHS cohort, at enrollment, an in-person interview was administered to all participants using a structured questionnaire. Baseline information included demographics, weight, height, cigarette smoking (which included smoking status, dosage, frequency, and age at starting to smoke for ever smokers, and age at quitting for former smokers), alcohol consumption (frequency and portion size), physical activity, and medical history such as physician-diagnosed diabetes and hypertension. We used a 165-item validated semiquantitative FFQ to record participants’ habitual diet during the past year at enrollment. The details regarding the development and validation of the FFQ were reported previously (18). Briefly, for each item, the FFQ included 8 categories of food intake frequencies (ranging from “never or hardly ever” to “two or more times a day”) and 3 portion sizes (small, medium, and large) for participants to choose from. We defined 2 diet patterns—“vegetable, fruit, and soy rich pattern” and “meat and dim sum pattern”—which were defined via principal component analysis and associated with the risk of type 2 diabetes in the SCHS cohort (12, 19). For physical activity, participants were asked for the average number of hours per week in the last year spent separately on *1*) moderate activity such as brisk walking, bowling, bicycling on level ground, and tai chi or chi kung, *2*) vigorous work such as moving heavy furniture, loading or unloading trucks, shoveling, or equivalent manual labor, and *3*) strenuous sports such as jogging, bicycling on hills, tennis, squash, swimming laps, or aerobics. There were 8 options provided for the response to each group of activities: never, 0.5–1 h, 2–3 h, 4–6 h, 7–10 h, 11–20 h, 21–30 h, and ≥31 h (23). We summed the total hours spent on physical activities for each participant, and created categories of <0.5 h/wk, 0.5 to <4 h/wk, and ≥4 h/wk for analysis. The height and weight were self-reported and the computation of BMI was the same as that used for the CKB cohort. A total of 16% of participants did not report either weight or height, and their BMI was calculated using imputed weight or height obtained from the linear regression equation: weight = *y*-intercept + gradient × height, where values for the *y*-intercept and gradient were derived from gender-specific weight-height regression lines obtained from all subjects with known heights and weights. If they reported neither weight nor height, their BMI could not be calculated. This method of data imputation was described in detail previously (24).

### Lifestyle score

Details of the lifestyle score in the 2 cohorts are described in **Supplemental Tables 1 and 2**. We summed each item of lifestyle factors in each cohort. The lifestyle score ranged from 0 to 29 in the CKB cohort and 0 to 17 in the SCHS cohort. We defined a healthy lifestyle as one associated with normal BMI and WHR, no smoking, moderate alcohol intakes, a high level of physical activities, and healthy diets (high consumption of vegetables, fruits, and whole grain, and low consumption of meat). The lower the lifestyle score, the more it was in line with a more healthy lifestyle.

### Single nucleotide polymorphisms and genotyping

For each participant, we collected a 10-mL nonfasting blood sample (with time of last meal recorded) into 1 EDTA-coated vacutainer (BD Hemogard^TM^). The samples were then kept in a portable, insulated cool box with ice packs (to maintain their temperature at –48°C) for up to a few hours before being taken to the local study laboratory for immediate processing. Single nucleotide polymorphisms (SNPs) for type 2 diabetes were genotyped in 95,680 randomly selected individuals using a 384-SNP Illumina GoldenGate® array and 32,410 participants were genotyped by a custom Affymetrix Axiom® 700K variant array (25). After exclusion of 2539 individuals based on call rate <98%, sex mismatch, heterozygosity *F* statistic SD score ≥5, Hardy–Weinberg disequilibrium (*P *< 0.05/384 = 1.3 × 10^−4^), or duplication of genetic data (*n* = 14,419), we included 111,132 participants with genetic data in the CKB cohort.

We genotyped 18,114 SCHS samples by nonfasting blood sample on the Illumina Global Screening Array version 1.0 and used this as the discovery data set in the study. A total of 2999 SCHS samples were genotyped on the Illumina Global Screening Array version 2.0 and utilized in the replication stage. After exclusion of individuals with call rates <95.0%, whose samples had extremes in heterozygosity, and whose samples were outliers, 16,779 SCHS samples genotyped on the Illumina Global Screening Array version 1.0 and 2705 SCHS samples genotyped on the Illumina Global Screening Array version 2.0 passed quality control procedures and were available for subsequent statistical analysis.

### Calculation of GRS

We derived the GRS based on the 49 SNPs in the CKB cohort and the 37 SNPs in the SCHS cohort. All SNPs were selected from previous GWASs for type 2 diabetes (25). We calculated the GRS for each individual as the sum of risk alleles at the selected SNPs. Three GRSs including diabetes genetic risk score (DM-GRS), β-cell genetic risk score (BC-GRS), and insulin resistance genetic risk score (IR-GRS) were calculated based on their pathophysiological mechanisms related to β-cell dysfunction and insulin resistance (25–29) (**Supplemental Table 3**). We assigned the mean genotype for that participant's region to impute missing genotypes (25).

### Ascertainment of type 2 diabetes

In both the CKB study and SCHS study, the primary endpoint is type 2 diabetes. In the CKB study, we used linkage with local disease, death registries, and the recently established national health insurance system to identify incident diabetes since participants were enrolled into the CKB study at baseline (18). All cases were coded with the 10th revision of the International Classification of Diseases by trained staff blinded to the baseline information. For our analysis, diabetes cases coded as E11 and E14 were included and we excluded other cases clearly defined as non–type 2 diabetes. Misclassification of other types of diabetes was almost impossible because the age of the participants ranged from 40 to 79 y, among whom the possibility of types 1 diabetes was very low. Besides, the incidence of other types of diabetes is lower than that of type 2 diabetes. During 2012–2013, clinical researchers in the Oxford International Coordinating Center of the CKB adjudicated the validity of the reported diabetes diagnoses in a random sample of 831 cases by reviewing their medical records; the accuracy rate was found to be 98.6% (14).

In the SCHS cohort, ascertainment of incident type 2 diabetes was done by asking the participants for a history of physician-diagnosed diabetes at baseline and both follow-up interviews using the question: “Have you been told by a doctor that you have diabetes?” If the answer was “yes,” participants were also asked for the age at which they were first diagnosed. Participants were classified as incident type 2 diabetes cases if they did not report diabetes at the initial baseline interview, and reported developing diabetes between the baseline interview and subsequent follow-up telephone interviews. The accuracy of self-reported diabetes, which was estimated by a separate study, was 98.8% in this cohort (12).

### Statistical analysis

We carried out individual participant data analyses in the present study. For the lifestyle association analysis, we included 461,030 participants without diagnosed diseases (diabetes, cancer, stroke, and coronary artery disease) or missing data in the CKB cohort and 38,434 participants without BMI missing in the SCHS cohort. Finally, we included 499,464 participants in the lifestyle association analysis (**Supplemental Figure 1**).

For the genetic association analysis, we excluded participants (*n* = 412,716) who did not have genetic (*n* = 401,759) or BMI data (*n* = 1), whose age was >79 y when last interviewing (*n*  = 28), and who had previously diagnosed cancer, coronary artery disease, stroke, and diabetes at baseline (*n* = 10,928) in the CKB cohort. Then 100,175 participants were included for genetic analysis. In the SCHS cohort, we excluded participants (*n* = 45,411) who were ineligible (*n* = 17,846), without BMI (*n* = 6977), or without genetic data (*n* = 22,262). A total of 16,172 participants were included in the final genetic data set. Finally, we included 116,347 participants in the genetic association analysis (Supplemental Figure 1).

With time-on-study as timescale, we calculated person-years for each participant from baseline entry date to the date of diagnosis of diabetes, death, loss to follow-up, or 31 December 2016 in the CKB cohort or last follow-up interview in the SCHS cohort, whichever came first.

A Cox proportional-hazards model was used to estimate the associations of genetic and lifestyle factors with incident type 2 diabetes, adjusted for age, sex, region code, and family history of diabetes in the CKB and adjusted for sex, age, education, father dialect, and years of interview in the SCHS. The participants in the 2 cohorts were pooled by quintiles of scores and the model was adjusted for sex, age, region code, and data sources. For the analysis of incident type 2 diabetes, each cohort was divided into 3 genetic or lifestyle risk groups: low/healthy (in the bottom tertile of the GRS/lifestyle score), middle/intermediate (in the middle tertile of the GRS/lifestyle score), and high/unhealthy (in the top tertile of the GRS/lifestyle score). We compared HRs for participants adhering to a healthy lifestyle or at a high genetic risk with those adhering to an unhealthy lifestyle or at a low genetic risk, respectively.

According to the DM-GRS and the lifestyle score, the participants were divided into 9 groups in the analysis of joint effect; participants at the highest genetic risk with the most unhealthy lifestyle served as the reference group. We also used Cox regression to calculate 10-y type 2 diabetes event rates, which were standardized to the mean of age, sex, region, and data sources within the study population.

The model of interaction between a single SNP and the lifestyle score was adjusted as previously mentioned in the CKB and SCHS cohorts. It was different from the interplay between a single lifestyle factor and DM-GRS, where we adjusted for sex, age, region code, diet (fruits, vegetables, meat, and whole grain), alcohol (nondrinker and current drinker), smoking (nonsmoker and current smoker), physical activity, BMI, data sources, and family history of diabetes in the CKB and sex, age, father dialect, years of interview, vegetable-fruit-soy pattern, meat-dim-sum pattern, alcohol (nondrinker and current drinker), smoking (nonsmoker and current smoker), physical activity, and BMI in the SCHS. We used the likelihood ratio test to compare models with and without cross-product terms to test the interaction. In subgroup analyses, we also estimated the joint effect stratified by demographic factors and lifestyle factors. All statistical analyses were performed using Stata version 15.1 (StataCorp). A 2-sided *P* value <0.05 was the threshold for statistical significance.

## Results

### Characteristics of participants in the CKB and SCHS cohorts

In the 2 prospective cohort studies, we included 512,891 participants in the CKB and 45,411 participants in the SCHS for lifestyle association analysis. The genetic analysis included 100,175 participants and 16,172 participants in the CKB and SCHS, respectively (**Table 1**). For genetic analysis, 3383 type 2 diabetes events were observed in the CKB (median follow-up: 9.67 y) and 2036 type 2 diabetes events in the SCHS (median follow-up: 10.73 y). In the pooled cohorts, we observed 19,514 type 2 diabetes events.

**TABLE 1:** Characteristics of the participants at baseline^1^

	CKB		SCHS	
Characteristic	Participants with genetic data (*n* = 100,175)	All participants (*n* = 512,891)	Participants with genetic data (*n* = 16,172)	All participants (*n* = 45,411)
Age	51.6 ± 10.8	51.5 ± 10.7	54.5 ± 7.2	55.2 ± 7.6
Male	42,128 (42.1)	210,259 (41.0)	7046 (43.6)	19,409 (42.7)
Smoking				
Never or occasional smoker	66,344 (66.2)	346,773 (67.6)	11,777 (72.8)	32,731 (72.1)
Ex-smoker	5854 (5.8)	30,563 (6.0)	1568 (9.7)	4311 (9.5)
Current smoker: 1–9 cigarettes/d	5737 (5.7)	26,794 (5.2)	1100 (6.8)	3387 (7.5)
Current smoker: 10–19 cigarettes/d	7736 (7.8)	37,138 (7.2)	1219 (7.5)	3502 (7.7)
Current smoker: ≥20 cigarettes/d	14,505 (14.5)	71,623 (14.0)	508 (3.1)	1480 (3.3)
Alcohol				
Men: 10–25 g/d; Women: 5–15 g/d	3277 (3.4)	17,053 (3.3)	517 (3.2)	1447 (3.2)
BMI, kg/m^2^	23.4 ± 3.4	23.7 ± 3.4	23.0 ± 3.4	23.0 ± 3.5
Lifestyle score				
Healthy	25.0	25.2	31.5	26.5
Intermediate	36.2	36.1	33.5	30.9
Unhealthy	38.8	38.7	35.0	42.7
DM-GRS				
Low	33.2	NA	29.5	NA
Middle	30.4	NA	32.4	NA
High	36.4	NA	38.2	NA

1 Values are means ± SDs, *n* (%), or percentages. The healthy lifestyle is the lowest tertile of lifestyle score. CKB, China Kadoorie Biobank; DM-GRS, diabetes genetic risk score; NA, not available; SCHS, Singapore Chinese Health Study.

### Associations of lifestyle with incidence of type 2 diabetes

Lifestyle score obeyed a normal distribution within the 2 cohorts (**Supplemental Figures 2, 3**). Participants with an unhealthy lifestyle had more obesity and were more likely to smoke, drink responsibly, exercise infrequently, and eat less fruits, vegetables, whole grains, and more meat in both cohorts (**Supplemental Tables 4, 5**). Our study showed that a higher lifestyle score was significantly associated with a higher risk of type 2 diabetes with a dose–response relation within each cohort (**Supplemental Figures 4, 5**). We observed that participants in the bottom quintile of the lifestyle score (a healthy lifestyle), as compared with participants in the top quintile of the lifestyle score (an unhealthy lifestyle), were at significantly lower risk of type 2 diabetes, with adjusted HRs of 0.30 (95% CI: 0.28, 0.33) in the CKB, 0.41 (95% CI: 0.37, 0.46) in the SCHS, and 0.30 (95% CI: 0.28, 0.32) in the pooled cohorts (**Table 2**). We also calculated the adjusted cumulative type 2 diabetes events rates stratified by lifestyle score; similar patterns were observed. Compared with an unhealthy lifestyle, a healthy lifestyle was associated with a lower type 2 diabetes events rate, with an adjusted HR of 0.38 (95% CI: 0.36, 0.40) in the CKB cohort, 0.49 (95% CI: 0.44, 0.55) in the SCHS cohort, and 0.41 (95% CI: 0.39, 0.43) in the pooled cohorts (**Supplemental Figures 6–8**).

**TABLE 2:** Association between quintile of lifestyle score and the risk of type 2 diabetes^1^

	Continuous score (total)	Quintile 1 (lowest)	Quintile 2	Quintile 3	Quintile 4	Quintile 5 (highest)	*P*-trend
CKB							
Person-years	4,551,091	722,620	998,084	568,464	1,041,943	1,219,980	
Type 2 diabetes cases, *n*	15,118	953	2185	1637	3996	6347	
Mean (range)	12.7 (0.0–29.0)	8.0 (0.0–9.0)	10.5 (10.0–11.0)	12.0 (12.0–12.0)	13.5 (13.0–14.0)	16.6 (15.0–29.0)	
Age adjusted	1.31 (1.29, 1.33)	0.31 (0.29, 0.33)	0.47 (0.45, 0.50)	0.59 (0.56, 0.62)	0.76 (0.73, 0.79)	1.00	<0.001
Multivariate adjusted	1.34 (1.32, 1.36)	0.30 (0.28, 0.33)	0.42 (0.40, 0.44)	0.53 (0.50, 0.56)	0.69 (0.66, 0.72)	1.00	<0.001
SCHS							
Person-years	105,078	18,617	14,184	16,541	31,379	24,357	
Type 2 diabetes cases, *n*	4392	476	474	591	1397	1454	
Mean (range)	8.8 (1.0–18.0)	3.3 (0.0–4.0)	5.0 (5.0–5.0)	6.0 (6.0–6.0)	7.5 (7.0–8.0)	10.1 (9.0–16.0)	
Age adjusted	1.14 (1.13, 1.16)	0.42 (0.38, 0.47)	0.55 (0.49, 0.62)	0.60 (0.54, 0.67)	0.74 (0.68, 0.80)	1.00	<0.001
Multivariate adjusted	1.15 (1.14, 1.17)	0.41 (0.37, 0.46)	0.54 (0.48, 0.60)	0.59 (0.53, 0.66)	0.72 (0.67, 0.79)	1.00	<0.001
Pooled							
Type 2 diabetes cases, *n*	19,510	1429	2659	2228	5393	7801	
Age adjusted		0.34 (0.32, 0.36)	0.47 (0.45, 0.49)	0.62 (0.59, 0.65)	0.79 (0.76, 0.82)	1.00	<0.001
Multivariate adjusted		0.30 (0.28, 0.32)	0.42 (0.40, 0.44)	0.53 (0.50, 0.56)	0.69 (0.66, 0.72)	1.00	<0.001

1 Values are HRs (95% CIs) unless otherwise indicated. We included all participants in the 2 studies when we estimated the association between lifestyle score and type 2 diabetes (CKB: 461,030; SCHS: 38,434). Model was adjusted for sex, age, region code, and family history of diabetes in the CKB cohort and adjusted for sex, age, education, father dialect, and years of interview in the SCHS cohort. The participants in the 2 cohorts were pooled by quintile of lifestyle score and the model was adjusted for sex, age, and region. Those in the highest quintile of lifestyle score serve as the reference group. CKB, China Kadoorie Biobank; SCHS, Singapore Chinese Health Study.

### Genetic associations with incidence of type 2 diabetes

GRSs obeyed a normal distribution within the 2 cohorts (Supplemental Figures 2, 3). As hypothesized, more participants at high genetic risk were inclined to less consumption of whole grains and more consumption of meats in the CKB (**Supplemental Table 6**). However, we did not observe any association in the SCHS (**Supplemental Table 7**). In addition, we found that a higher GRS was associated with a higher risk of type 2 diabetes (Supplemental Figures 4, 5). The risk of type 2 diabetes was 79% higher among participants at the highest genetic risk than among those at the lowest genetic risk in the CKB (HR: 1.79; 95% CI: 1.60, 2.00), 106% higher in the SCHS (HR: 2.06; 95% CI: 1.74, 2.41), and 90% higher in the pooled cohorts (HR: 1.90; 95% CI: 1.74, 2.08). The association between BC-GRS and type 2 diabetes was similar to DM-GRS, but not IR-GRS (**Table 3**, **Supplemental Table 8**). Relative to those at low genetic risk, participants at high genetic risk had a 1.50 (95% CI: 1.38, 1.63) higher type 2 diabetes events rate in the CKB, 1.65 (95% CI: 1.48, 1.84) in the SCHS, and 1.57 (95% CI: 1.47, 1.67) in the pooled cohorts (Supplemental Figures 6–8).

**TABLE 3:** Association between quintile of diabetes GRS and the risk of type 2 diabetes^1^

	Continuous score (total)	Quintile 1 (lowest)	Quintile 2	Quintile 3	Quintile 4	Quintile 5 (highest)	*P*-trend
CKB							
Person-years	968,851	183,260	154,713	185,713	250,560	194,605	
Type 2 diabetes cases, *n*	3383	478	498	617	932	858	
Mean (range)	51.2 (34.0–71.0)	45.4 (34.0–47.9)	48.6 (48.0–49.9)	50.6 (50.0–51.9)	53.0 (52.0–54.9)	56.8 (55.0–71.0)	
Age adjusted	1.13 (1.10, 1.16)	1.00	1.24 (1.09, 1.40)	1.28 (1.14, 1.44)	1.45 (1.29, 1.61)	1.72 (1.54, 1.92)	<0.001
Multivariate adjusted	1.14 (1.12, 1.17)	1.00	1.25 (1.10, 1.42)	1.30 (1.15, 1.46)	1.48 (1.32, 1.65)	1.79 (1.60, 2.00)	<0.001
SCHS							
Person-years	173,539	24,265	45,210	20,429	35,451	48,184	
Type 2 diabetes cases, *n*	2036	184	472	207	428	745	
Mean (range)	28.4 (25.0–32.0)	32.7 (25.0–34.9)	36.1 (35.0–37.9)	38.0 (38.0–38.9)	39.5 (39.0–40.9)	42.7 (41.0–52.0)	
Age adjusted	1.06 (1.05, 1.07)	1.00	1.39 (1.17, 1.64)	1.34 (1.10, 1.64)	1.60 (1.35, 1.90)	2.05 (1.74, 2.41)	<0.001
Multivariate adjusted	1.06 (1.05, 1.08)	1.00	1.39 (1.17, 1.64)	1.34 (1.10, 1.63)	1.59 (1.34, 1.89)	2.06 (1.75, 2.42)	<0.001
Pooled							
Type 2 diabetes cases, *n*	5419	662	970	824	1360	1603	
Age adjusted		1.00	1.47 (1.34, 1.63)	1.27 (1.15, 1.41)	1.51 (1.37, 1.65)	2.04 (1.87, 2.24)	<0.001
Multivariate adjusted		1.00	1.30 (1.17, 1.43)	1.32 (1.19, 1.46)	1.52 (1.39, 1.67)	1.90 (1.74, 2.08)	<0.001

1 Values are HRs (95% CIs) unless otherwise indicated. We included all participants in the 2 studies when we estimated the association between lifestyle score and type 2 diabetes (CKB: 461,030; SCHS: 38,434). Model was adjusted for sex, age, region code, data sources (genetic data from genome-wide association study or single nucleotide polymorphism panel), and family history of diabetes in the CKB cohort and adjusted for sex, age, education, father dialect, and years of interview in the SCHS cohort. The participants in the 2 cohorts were pooled by quintile of GRS and the model was adjusted for sex, age, region, and data sources. Those in the lowest quintile of genetic risk serve as the reference group. CKB, China Kadoorie Biobank; GRS, genetic risk score; SCHS, Singapore Chinese Health Study.

### Genetic risk, lifestyle, and type 2 diabetes

**FIGURE 1:**
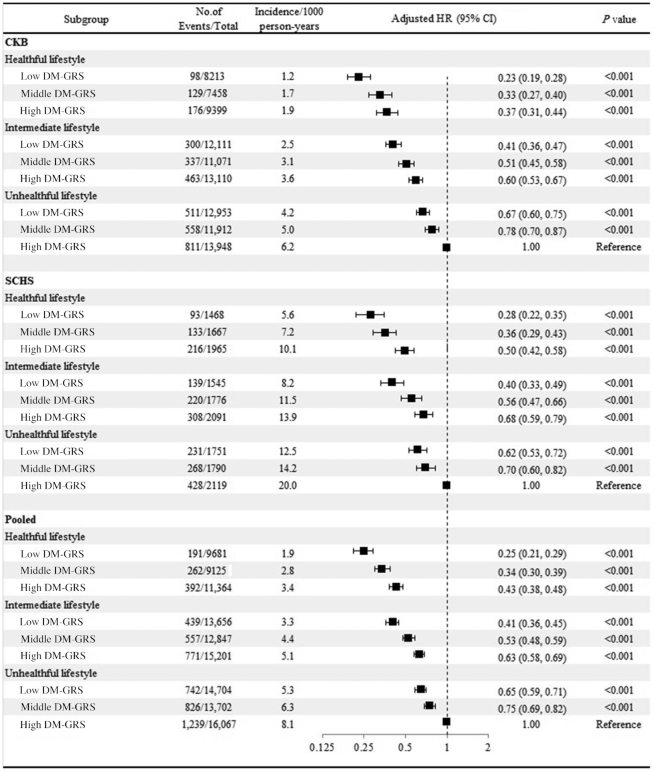
Adjusted HRs for type 2 diabetes events, according to DM-GRS and lifestyle score. In these comparisons, participants with a low DM-GRS and healthy lifestyle serve as the reference group. The participants in the 2 cohorts were pooled and the model was adjusted for sex, age, region, and data sources. There was no evidence of significant interactions between genetic and lifestyle risk factors (*P*-interaction = 0.60 in the pooled cohort, 0.38 in the CKB, 0.10 in the SCHS). Unadjusted incidence rates are reported per 1000 person-years of follow-up. CKB, China Kadoorie Biobank; DM-GRS, diabetes genetic risk score; SCHS, Singapore Chinese Health Study.

**FIGURE 2:**
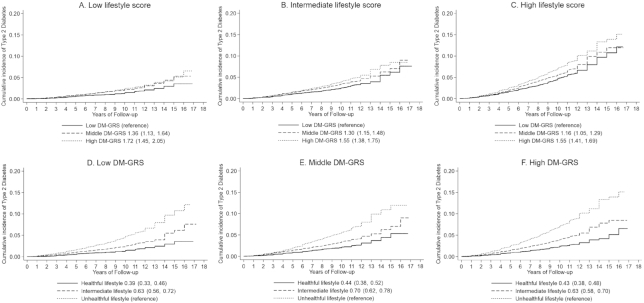
Adjusted type 2 diabetes events rates, stratified by lifestyle score (A–C) and DM-GRS (D–F), of participants in the pooled cohort. The 95% CIs for the HRs are provided in parentheses. The participants in the 2 cohorts were pooled by tertile of lifestyle score or genetic risk within each cohort. Cox regression models were adjusted for age, sex, region, and data source, which was performed on cohort-specific population averages for each covariate. DM-GRS, diabetes genetic risk score.

In the CKB, we found that a healthy lifestyle was associated with a lower risk of type 2 diabetes within each category of genetic risk (**Figure 1**). As compared with a high genetic risk and an unhealthy lifestyle, an intermediate lifestyle was associated with a 40% (95% CI: 33%, 47%) lower risk of type 2 diabetes among those at a high genetic risk and a healthy lifestyle was associated with a 63% (95% CI: 56%, 69%) lower risk of type 2 diabetes among those at a high genetic risk in the CKB. We successfully replicated the results in the SCHS. In the pooled cohorts, as compared with a high genetic risk and an unhealthy lifestyle, a healthy lifestyle was associated with a 57% (95% CI: 52%, 62%) lower risk of type 2 diabetes among those at a high genetic risk, 66% (95% CI: 61%, 70%) lower among those at a middle genetic risk, and 75% (95% CI: 71%, 79%) lower among those at a low genetic risk. Similarly, the participants with a healthy lifestyle showed significantly lower adjusted cumulative type 2 diabetes event rates than those with an unhealthy lifestyle, with an adjusted HR of 0.39 (95% CI: 0.33, 0.46) among participants at a low genetic risk, 0.44 (95% CI: 0.38, 0.52) among participants at a middle genetic risk, and 0.43 (95% CI: 0.38, 0.48) among participants at a high genetic risk, respectively (**Figure 2**).

Moreover, we did not find significant interactions between single SNPs and lifestyle score in the 2 cohorts (**Supplemental Tables 9, 10**). However, the interplay of WHR in the CKB cohort, and of smoking and BMI in the SCHS cohort with genetic risk were statistically significant (**Supplemental Tables 11, 12**). In subgroup analyses, we did not find sex-specific effects, and the joint effect results were consistent with the results in the total population (**Supplemental Tables 13, 14**).

### The standardized 10-y type 2 diabetes rates according to genetic and lifestyle risk

**FIGURE 3:**
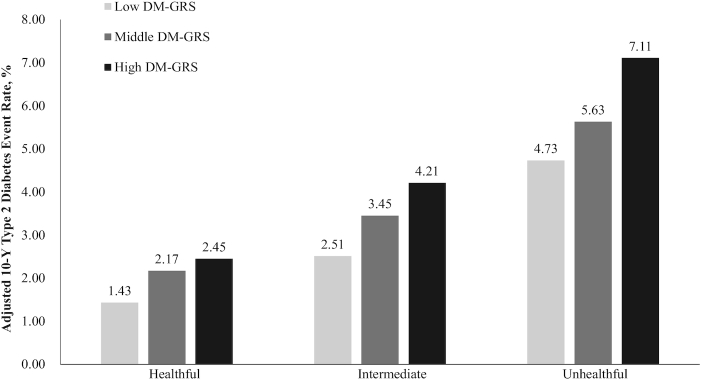
Adjusted 10-y cumulative type 2 diabetes event rates in the pooled cohort, according to lifestyle score and DM-GRS, standardized to the means of age, sex, region, and data sources within the study population. DM-GRS, diabetes genetic risk score.

Among participants with an unhealthy lifestyle, the standardized 10-y type 2 diabetes rates were 5.45% among those at low genetic risk and 8.47% among those at high genetic risk in the CKB cohort, 10.87% and 17.73% in the SCHS cohort, and 4.73% and 7.11% in the pooled cohort, respectively. Among participants at high genetic risk, the standardized 10-y type 2 diabetes rates were 2.66% among those with a healthy lifestyle in the CKB cohort, 9.34% in the SCHS cohort, and 2.45% in the pooled cohort (**Figure 3**, **Supplemental Figures 9, 10**). We observed a similar trend in the BC-GRS, but the trend in the IR-GRS was nonsignificant (**Supplemental Figures 11–14**).

## Discussion

To the best of our knowledge, the present individual participant data analyses of 2 independent prospective cohorts is the largest study to date providing quantitative data about genetic risk, lifestyle risk, and their interactions on the risk of type 2 diabetes. Among 0.55 million Chinese adults, high genetic risk was independent of lifestyle behaviors and was associated with a higher risk of type 2 diabetes. Within any genetic risk category, a healthy lifestyle was associated with a significantly decreased risk of type 2 diabetes among the Chinese population.

Our findings have 3 implications significant for public health. First, our study indicated that the effect of genetic risk was independent of traditionally measured lifestyle factors. The observed robust genetic associations with a higher risk of type 2 diabetes in the present study were well aligned with previous genetic studies of white populations (16, 17), where each risk allele was associated with an ∼16–19% higher risk of type 2 diabetes.

Second, our findings showed that a healthy lifestyle was associated with a lower risk of type 2 diabetes regardless of genetic risk, sex, age, and region, consistent with previous observations in white populations from the United States (30) and Europe (31). The replicable findings from the 2 independent nationally representative cohorts provided strong evidence for the beneficial effects of adhering to a healthy lifestyle on the development of type 2 diabetes in Chinese populations.

Third, our data showed that the absolute risk associated with a healthy lifestyle decreased within each category of genetic risk. The joint effects between genetic risk and lifestyle factors were in line with the evidence from Western studies. For example, the genetic risk of type 2 diabetes was modified by the Western dietary pattern and obesity in US health professional cohorts (15, 17). Our findings suggest that we are supposed to encourage the whole Chinese population to adhere to a healthy lifestyle, regardless of their genes, to reduce the risk of type 2 diabetes.

The mechanisms behind the observed results are not fully understood. However, our findings were biologically plausible. Previous evidence from Mendelian randomization analyses implied that BMI (32), WHR (33), diets (34), and smoking (34) as components of lifestyle were causally associated with type 2 diabetes. Besides, it is worth noting that we observed interactions between genetic risk and WHR or BMI on risk of type 2 diabetes, in agreement with results in Western populations (16, 17). Taken together, these findings might at least partially support our results. However, we cannot exclude the influence of other biological pathways, and future related researches are needed to provide biological insights into the joint effects between genetic risk and lifestyle factors on the risk of type 2 diabetes among Chinese adults.

Several strengths merit consideration. First, our study, to our knowledge for the first time, provided evidence for the joint effects of genetic risk and multiple lifestyle factors on the risk of type 2 diabetes among Chinese from 2 independent nationally representative cohorts. Second, a standardized analysis strategy was used to analyze individual participant data from the 2 cohorts. This method might mitigate differences in statistical methods and improve the overall reliability of the results, as well as allowing us to adjust for the same set of covariates across studies. Our sample size was very large, which improved the power of our analysis. Third, the inclusion of a geographically spread population living in 2 countries, with different sociodemographic characteristics, makes our results widely generalizable and representative to examine interactions because of the greater variability of lifestyle factors. Fourth, genotyping was performed with high-quality control standards within 2 cohorts (25) so we could guarantee the accuracy of the genetic data as far as possible. Fifth, we controlled for potential confounding factors and sought to minimize the reverse causation bias by excluding participants with major chronic diseases at baseline, which might lead to lifestyle changes. Sixth, detailed collection of dietary data through face-to-face interviews used an FFQ that was specifically developed and validated in 2 cohorts (19, 20). The anthropometric information was measured by trained staff rather than self-reported, thus providing more accurate estimates of BMI and WHR.

Our study also has several limitations. First, we only included 49 SNPs in the CKB and 37 SNPs in the SCHS to calculate the DM-GRS, which were only small proportions of the SNPs related to diabetes. However, most of the SNPs associated with diabetes were based on the Western population (7); also, our cohorts were established >10 y ago. We had difficulty including all related SNPs in our genetic database. In addition, the GRS in the SCHS cohort was a little different from that in the CKB cohort because 10 SNPs were not included in the SCHS cohort. However, we examined the association between GRS and type 2 diabetes in each cohort, which showed little difference. Second, participants in each cohort used slightly different methods to assess lifestyle at baseline. Moreover, trained staff measured the lifestyle factors once at baseline and thus this might not necessarily reflect long-term exposures. Third, the lifestyle scores in the 2 cohorts were defined differently, but our results were consistent within each cohort. Fourth, on account of a lack of comprehensive assessment of food consumption, we were not able to capture the complexity of the dietary patterns. Residual confounding may have existed in our study. Fifth, we could not collect the data on medication among all participants, so it is difficult to ascertain the influence of medication in our study. Sixth, the identification of incident diabetes relied on the health insurance system in the CKB cohort and questionnaire in the SCHS cohort, but some cases of asymptomatic diabetes might have been undiagnosed. Seventh, population stratification was likely to lead to bias. However, almost all of the participants we surveyed in our cohorts were Han people. The impact of population stratification was minimal in our analysis. Finally, nondifferential misclassification was likely to exist in our study, which might lead to attenuation of effect estimates.

In conclusion, our study combined 2 large prospective cohorts of China and Singapore to provide quantitative estimates of genetic and lifestyle risks of type 2 diabetes. Our findings suggest adherence to a healthy lifestyle was associated with substantially lower risk of type 2 diabetes regardless of genetic risk, although we did not observe a gene–lifestyle interaction on type 2 diabetes. Our study lends robust support to adopting a healthy lifestyle for the reduction of type 2 diabetes in the Chinese population.

## Supplementary Material

nqz310_Supplemental_Figures_and_Tables

## Notes

Supported by National Key R&D Program of China grants 2016YFC0900500, 2016YFC0900501, and 2016YFC0900504. The CKB baseline survey and the first re-survey were supported by a grant from the Kadoorie Charitable Foundation in Hong Kong. The long-term follow-up is supported by UK Wellcome Trust grants 202922/Z/16/Z, 088158/Z/09/Z, and 104085/Z/14/Z; National Natural Science Foundation of China grants 81390540, 81390544, and 81390541; and Chinese Ministry of Science and Technology grant 2011BAI09B01. The Singapore Chinese Health Study was supported by National Medical Research Council, Singapore grant NMRC/CIRG/1456/2016 and NIH grants R01 CA144034 and UM1 CA182876. W-PK was supported by National Medical Research Council, Singapore grant NMRC/CSA/0055/2013.

The funders had no role in the study design, data collection, data analysis and interpretation, writing of the report, or the decision to submit the manuscript for publication.

Supplemental Tables 1–14 and Supplemental Figures 1–14 are available from the “Supplementary data” link in the online posting of the article and from the same link in the online table of contents at https://academic.oup.com/ajcn/.

Data described in the article, code book, and analytic code will be made available from CKB upon request (https://www.ckbiobank.org/site/Data+Access) or from SCHS upon request pending application and approval. The data sets generated and/or analyzed during the current study are not publicly available but are available from the corresponding author on reasonable request.

Abbreviations used: BC-GRS, β-cell genetic risk score; CKB, China Kadoorie Biobank; DM-GRS, diabetes genetic risk score; GRS, genetic risk score; GWAS, genome-wide association study; IR-GRS, insulin resistance genetic risk score; MET, metabolic equivalent task; SCHS, Singapore Chinese Health Study; SNP, single nucleotide polymorphism; WHR, waist-to-hip ratio.
