# Supplementary material for: Genetic risk, adherence to a healthy lifestyle, and type 2 diabetes risk among 550,000 Chinese adults: results from 2 independent Asian cohorts
Source: Am J Clin Nutr. 2020 Jan 24;111(3):698–707. doi: 10.1093/ajcn/nqz310 (PMC7049535; doi:10.1093/ajcn/nqz310)
Supplement: nqz310_Supplemental_Figures_and_Tables [file nqz310_supplemental_figures_and_tables.docx]

**Online Supplementary Material**

Genetic risk, adherence to a healthful lifestyle, and type 2 diabetes risk among 550,000 Chinese adults: Results from two independent Asian cohorts

Fist Author: Haoxin Li

**Supplementary Table 1. Component of lifestyle score in the CKB^1^**

| **Lifestyle** | **Category** | **Score** |
| --- | --- | --- |
| BMI | <23.9 kg/m^2^ | 0 |
|  | 24.0-27.9 kg/m^2^ | 1 |
|  | ≥28.0 kg/m^2^ | 2 |
|  |  |  |
| Waist hip ratio | Men：<0.90；Women：<0.85 | 0 |
|  | Men：0.90-0.95；Women：0.85-0.90 | 1 |
|  | Men：≥0.95；Women：≥0.90 | 2 |
|  |  |  |
| Diet (fruits, vegetables and whole grain) | Daily | 0 |
|  | 4-6 days per week | 1 |
|  | 1-3 days per week | 2 |
|  | Monthly | 3 |
|  | Never/rarely | 4 |
|  |  |  |
| Diet (meats) | Never/rarely | 0 |
|  | Monthly | 1 |
|  | 1-3 days per week | 2 |
|  | 4-6 days per week | 3 |
|  | Daily | 4 |
|  |  |  |
| Physical activity  (MET hours/day) | Highest quintile | 0 |
|  | Second quintile | 1 |
|  | Third quintile | 2 |
|  | Fourth quintile | 3 |
|  | Lowest quintile | 4 |
|  |  |  |
| Alcohol | Men：10-25 g/d；Women：5-15 g/d | 0 |
|  | Others | 1 |
|  |  |  |
| Smoking | Never or occasional smoker | 0 |
|  | Ex-smoker | 1 |
|  | Current smoker: 1-9 cig/day | 2 |
|  | Current smoker: 10-19 cig/day | 3 |
|  | Current smoker: ≥20 cig/day | 4 |

^1^ Lifestyle score ranges from 0 to 29.

**Supplementary Table 2. Component of lifestyle score in the SCHS^1^**

| **Lifestyle** | **Category** | **Score** |
| --- | --- | --- |
| BMI | <20.0 kg/m^2^ | 0 |
|  | 20-22.9 kg/m^2^ | 1 |
|  | 23-24.9 kg/m^2^ | 2 |
|  | 25-27.4 kg/m^2^ | 3 |
|  | ≥27.5 kg/m^2^ | 4 |
|  |  |  |
| VFS | Highest quartile | 0 |
| (vegetable-fruit-soy pattern) | Second quartile | 1 |
|  | Third quartile | 2 |
|  | Lowest quartile | 3 |
|  |  |  |
| MDS | Lowest quartile | 0 |
| (meat-dim-sum pattern) | Second quartile | 1 |
|  | Third quartile | 2 |
|  | Highest quartile | 3 |
|  |  |  |
| Physical activity | 4+ h/wk | 0 |
|  | 0.5-<4 h/wk | 1 |
|  | <0.5 h/wk | 2 |
|  |  |  |
| Alcohol | Men：10-25 g/d；Women：5-15 g/d | 0 |
|  | Others | 1 |
|  |  |  |
| Smoking | Never or occasional smoker | 0 |
|  | Ex-smoker | 1 |
|  | Current smoker: 1-12 cig/day | 2 |
|  | Current smoker: 13-22 cig/day | 3 |
|  | Current smoker: 23+ cig/day | 4 |

^1^ Lifestyle score ranges from 0 to 17.

**Supplementary Table 3. Genotype information in the study^1^**

| **SNP** | **Chr** | **Risk allele** | **Other allele** | **Nearby gene** | **GWAS study reported OR** | **Classification** | **Risk allele frequency in the CKB** | **Risk allele frequency in the SCHS** |
| --- | --- | --- | --- | --- | --- | --- | --- | --- |
| rs340874 | 1 | C | T | *PROX1* | 1.07 | Beta Cell Function | 0.392 | 0.405 |
| rs7578597 | 2 | T | C | *THADA* | 1.15 | Beta Cell Function | 0.993 | 0.996 |
| rs11708067 | 3 | A | G | *ADCY5* | 1.12 | Beta Cell Function | 0.997 | 0.998 |
| rs1470579 | 3 | C | A | *IGF2BP2* | 1.14 | Beta Cell Function | 0.257 | 0.234 |
| rs16861329 | 3 | C | T | *ST64GAL1* | 1.09 | Beta Cell Function | 0.809 | 0.796 |
| rs6815464 | 4 | C | G | *MAEA* | 1.13 | Beta Cell Function | 0.442 | 0.554 |
| rs7754840 | 6 | C | G | *CDKAL1* | 1.12 | Beta Cell Function | 0.409 | 0.366 |
| rs4607517 | 7 | A | G | *GCK* | 1.07 | Beta Cell Function | 0.210 | 0.194 |
| rs6467136 | 7 | G | A | *GCC1-PAX4* | 1.11 | Beta Cell Function | 0.784 | 0.807 |
| rs13266634 | 8 | C | T | *SLC30A8* | 1.15 | Beta Cell Function | 0.534 | 0.543 |
| rs7041847 | 9 | A | G | *GLIS3* | 1.10 | Beta Cell Function | 0.463 | 0.480 |
| rs10811661 | 9 | T | C | *CDKN2A/B* | 1.20 | Beta Cell Function | 0.544 | 0.578 |
| rs1111875 | 10 | C | T | *HHEX/IDE* | 1.13 | Beta Cell Function | 0.280 | N.A |
| rs7901695 | 10 | C | T | *TCF7L2* | 1.41 | Beta Cell Function | 0.033 | 0.022 |
| rs4752781 | 11 | T | A | *DUSP8/INS* | 1.35 | Beta Cell Function | 0.833 | N.A |
| rs2237892 | 11 | C | T | *KCNQ1* | 1.40 | Beta Cell Function | 0.677 | 0.664 |
| rs5215 | 11 | C | T | *KCNJ11* | 1.14 | Beta Cell Function | 0.385 | 0.346 |
| rs1552224 | 11 | A | C | *ARAP1* | 1.14 | Beta Cell Function | 0.916 | 0.934 |
| rs10830963 | 11 | G | C | *MTNR1B* | 1.09 | Beta Cell Function | 0.428 | 0.436 |
| rs1359790 | 13 | G | A | *SPRY2* | 1.15 | Beta Cell Function | 0.716 | 0.711 |
| rs7172432 | 15 | A | G | *VPS13C* | 1.11 | Beta Cell Function | 0.618 | 0.669 |
| rs2028299 | 15 | C | A | *AP3S2* | 1.10 | Beta Cell Function | 0.202 | 0.195 |
| rs8042680 | 15 | A | C | *PRC1* | 1.07 | Beta Cell Function | 0.990 | N.A |
| rs4430796 | 17 | G | A | *HNF1B* | 1.14 | Beta Cell Function | 0.279 | 0.264 |
| rs6017317 | 20 | G | T | *HNF4A* | 1.09 | Beta Cell Function | 0.426 | 0.417 |
| rs780094 | 2 | C | T | *GCKR* | 1.06 | Insulin Resistance | 0.487 | 0.544 |
| rs7593730 | 2 | C | T | *RBMS1* | 1.11 | Insulin Resistance | 0.836 | 0.838 |
| rs3923113 | 2 | A | C | *GRB14* | 1.09 | Insulin Resistance | 0.866 | 0.865 |
| rs2943641 | 2 | C | T | *IRS1* | 1.19 | Insulin Resistance | 0.925 | 0.928 |
| rs831571 | 3 | C | T | *PSMD6* | 1.09 | Insulin Resistance | 0.633 | N.A |
| rs972283 | 7 | G | A | *KLF14* | 1.07 | Insulin Resistance | 0.712 | N.A |
| rs10923931 | 1 | T | G | *NOTCH2* | 1.13 | Other | 0.031 | N.A |
| rs243021 | 2 | A | G | *BCL11A* | 1.08 | Other | 0.670 | 0.661 |
| rs6780569 | 3 | G | A | *UBE2E2* | 1.21 | Other | 0.799 | N.A |
| rs4607103 | 3 | C | T | *ADAMTS9* | 1.09 | Other | 0.638 | 0.673 |
| rs4457053 | 5 | G | A | *ZBED3* | 1.08 | Other | 0.053 | 0.066 |
| rs9470794 | 6 | C | T | *ZFAND3* | 1.12 | Other | 0.317 | N.A |
| rs864745 | 7 | T | C | *JAZF1* | 1.10 | Other | 0.766 | N.A |
| rs896854 | 8 | T | C | *TP53INP1* | 1.06 | Other | 0.308 | N.A |
| rs17584499 | 9 | T | C | *PTPRD* | 1.57 | Other | 0.101 | 0.109 |
| rs13292136 | 9 | C | T | *TLE4/CHCHD9* | 1.11 | Other | 0.909 | 0.919 |
| rs10906115 | 10 | A | G | *CDC123* | 1.13 | Other | 0.626 | 0.606 |
| rs1802295 | 10 | A | G | *VPS26A* | 1.08 | Other | 0.109 | 0.116 |
| rs7961581 | 12 | C | T | *TSPAN8/LGR5* | 1.09 | Other | 0.215 | 0.226 |
| rs7178572 | 15 | G | A | *HMG20A* | 1.09 | Other | 0.349 | 0.330 |
| rs11634397 | 15 | G | A | *ZFAND6* | 1.06 | Other | 0.089 | 0.081 |
| rs9939609 | 16 | A | T | *FTO* | 1.15 | Other | 0.124 | N.A |
| rs4523957 | 17 | T | G | *SRR* | 1.28 | Other | 0.705 | 0.656 |
| rs5945326 | X | A | G | *DUSP9* | 1.27 | Other | 0.605 | N.A |

^1^ Classification criteria are as described in the main text. Genetic risk scores were constructed based on different sets of SNPs. If single SNP was not available in the SCHS, N.A is displayed. Missing values were imputed to the population mean.

**Supplementary Table 4. Baseline characteristics by lifestyle score category in the CKB^1^**

| **Characteristic** | **Healthful**  **(N= 116,871)** | **Intermediate**  **(N=** **167,369)** | **Unhealthful**  **(N=** **176,790)** | **P-value** |
| --- | --- | --- | --- | --- |
| Age (S.D) | 47.3 (9.6) | 50.6 (10.3) | 53.1 (10.6) | <0.001 |
| Male (%) | 27,479 (23.5) | 54,443 (32.5) | 107,178 (60.6) | <0.001 |
| Smoking (%) |  |  |  | <0.001 |
| Never or occasional smoker | 105,422 (90.2) | 130,235 (77.8) | 76,221 (43.1) |  |
| Ex-smoker | 3,935 (3.4) | 9,471 (5.7) | 11,262 (6.4) |  |
| Current smoker: 1-9 cig/day | 3,941 (3.4) | 8,976 (5.4) | 11,215 (6.3) |  |
| Current smoker: 10-19 cig/day | 1,974 (1.7) | 9,264 (5.5) | 22,714 (12.9) |  |
| Current smoker: ≥20 cig/day | 1,599 (1.4) | 9,423 (5.6) | 54,378 (31.3) |  |
| Alcohol (%) |  |  |  |  |
| Men：10-25 g/d；Women：5-15 g/d | 3,353 (2.9) | 5,138 (3.1) | 6,816 (3.9) | <0.001 |
| Fruits (%) |  |  |  |  |
| Never/rarely | 4,024 (3.4) | 6,896 (4.1) | 16,511 (9.3) | <0.001 |
| Monthly | 32,468 (27.8) | 56,077 (33.5) | 70,528 (39.9) |  |
| 1-3 days per week | 30,988 (26.5) | 54,948 (32.8) | 61,013 (34.5) |  |
| 4-6 days per week | 14,050 (12.0) | 17,564 (10.5) | 12,205 (6.9) |  |
| Daily | 35,341 (30.2) | 31,884 (19.1) | 16,533 (9.4) |  |
| Vegetables (%) |  |  |  |  |
| Never/rarely | 2 (0.0) | 12 (0.0) | 96 (0.1) | <0.001 |
| Monthly | 15 (0.1) | 453 (0.3) | 855 (0.5) |  |
| 1-3 days per week | 373 (0.3) | 2,267 (1.4) | 3,977 (2.3) |  |
| 4-6 days per week | 2,229 (1.9) | 6,590 (4.0) | 7,849 (4.4) |  |
| Daily | 114,252 (97.8) | 158,047 (94.4) | 164,013 (92.8) |  |
| Whole grains (%) |  |  |  |  |
| Never/rarely | 14,790 (12.7) | 44,058 (26.3) | 76,600 (43.3) | <0.001 |
| Monthly | 44,449 (38.0) | 81,263 (48.6) | 81,072 (45.9) |  |
| 1-3 days per week | 21,524 (18.4) | 20,899 (12.5) | 12,982 (7.3) |  |
| 4-6 days per week | 1,974 (1.7) | 1,113 (0.7) | 479 (0.3) |  |
| Daily | 34,134 (29.2) | 20,036 (12.0) | 5,657 (3.2) |  |
| Meats (%) |  |  |  |  |
| Never/rarely | 14,419 (12.3) | 5,750 (3.4) | 1,404 (0.8) | <0.001 |
| Monthly | 26,011 (22.3) | 22,460 (13.4) | 9,570 (5.4) |  |
| 1-3 days per week | 45,743 (39.1) | 65,975 (39.4) | 53,174 (30.1) |  |
| 4-6 days per week | 13,237 (11.3) | 31,126 (18.6) | 39,860 (22.6) |  |
| Daily | 17,461 (15.0) | 42,058 (25.1) | 72,782 (41.2) |  |
| Physical activity MET hours/day (S.D) | 28.6 (14.2) | 22.3 (13.3) | 16.9 (12.3) | <0.001 |
| Body mass index (S.D) | 22.5 (2.7) | 23.45 (3.3) | 24.3 (3.5) | <0.001 |
| Waist hip ratio (S.D) | 0.84 (0.1) | 0.87 (0.1) | 0.91 (0.1) | <0.001 |

^1^ P-values computed via ANOVA for continuous variables and chi-square test for categorical variables. We included all participants (N=461,030) when we described baseline characteristics in the CKB cohort.

**Supplementary Table 5. Baseline characteristics by lifestyle score category in the SCHS^1^**

| **Characteristic** | **Healthful**  **(N=11,881)** | **Intermediate**  **(N=12,213)** | **Unhealthful**  **(N=14,340)** | **P-value** |
| --- | --- | --- | --- | --- |
| Age (S.D) | 55.6 (7.7) | 54.6 (7.4) | 54.2 (7.3) | <0.001 |
| Male (%) | 3,980 (33.5) | 4,953 (40.6) | 7,881 (55.0) | <0.001 |
| Smoking (%) |  |  |  | <0.001 |
| Never or occasional smoker | 10,454 (88.0) | 9,445 (77.3) | 7,928 (55.3) |  |
| Ex-smoker | 841 (7.1) | 1,201 (9.8) | 1,701 (11.9) |  |
| Current smoker: 1-12 cig/day | 400 (3.4) | 848 (6.9) | 1,518 (10.6) |  |
| Current smoker: 13-22 cig/day | 174 (1.5) | 565 (4.6) | 2,135 (14.9) |  |
| Current smoker: 23+ cig/day | 12 (0.1) | 154 (1.3) | 1,058 (7.4) |  |
| Alcohol (%) |  |  |  |  |
| Men：10-25 g/d；Women：5-15 g/d | 366 (3.1) | 383 (3.1) | 509 (3.6) | 0.06 |
| Vegetable-fruit-soy pattern (S.D) | 2.1 (0.9) | 1.5 (1.1) | 0.9 (1.0) | <0.001 |
| Meat-dim-sum pattern (S.D) | 0.8 (0.9) | 1.5 (1.0) | 2.3 (0.9) | <0.001 |
| Physical activity h/wk (S.D) | 2.5 (4.9) | 1.7 (4.4) | 1.2 (4.0) | <0.001 |
| Body mass index (S.D) | 21.2 (2.5) | 22.8 (3.2) | 24.6 (1.7) | <0.001 |

^1^ P-values computed via ANOVA for continuous variables and chi-square test for categorical variables. We included all participants (N=38,434) when we described baseline characteristics in the SCHS cohort.

**Supplementary Table 6. Baseline characteristics by DM-GRS category in the CKB^1^**

| **Characteristic** | **Low DM-GRS (N=33,277)** | **Middle DM-GRS (N=30,441)** | **High DM-GRS (N=36,457)** | **P-value** |
| --- | --- | --- | --- | --- |
| Age (S.D) | 51.6 (10.8) | 51.8 (10.8) | 51.4 (10.8) | 0.003 |
| Male (%) | 13,963 (42.0) | 12,887 (42.3) | 15,277 (41.9) | 0.49 |
| Smoking (%) |  |  |  | 0.58 |
| Never or occasional smoker | 22,052 (66.3) | 20,135 (66.1) | 24,157 (66.3) |  |
| Ex-smoker | 1,978 (5.9) | 1,734 (5.7) | 2,142 (6.0) |  |
| Current smoker: 1-9 cig/day | 1,883 (5.7) | 1,804 (5.9) | 2,050 (5.6) |  |
| Current smoker: 10-19 cig/day | 2,585 (7.8) | 2,313 (7.6) | 2,837 (7.8) |  |
| Current smoker: ≥20 cig/day | 4,779 (14.3) | 4,455 (14.6) | 5,271 (14.5) |  |
| Alcohol (%) |  |  |  |  |
| Men：10-25 g/d；Women：5-15 g/d | 1,107 (3.3) | 1,010 (3.3) | 1,160 (3.2) | 0.48 |
| Fruits (%) |  |  |  |  |
| Never/rarely | 2,059 (6.2) | 2,040 (6.7) | 2,457 (6.7) | 0.01 |
| Monthly | 12,155 (36.5) | 11,175 (36.7) | 13,435 (36.9) |  |
| 1-3 days per week | 10,509 (31.6) | 9,587 (31.5) | 11,391 (31.3) |  |
| 4-6 days per week | 2,977 (8.9) | 2,680 (8.8) | 3,337 (9.2) |  |
| Daily | 5,577 (16.8) | 4,959 (16.3) | 5,837 (16.0) |  |
| Vegetables (%) |  |  |  |  |
| Never/rarely | 14 (0.1) | 8 (0.1) | 9 (0.1) | 0.71 |
| Monthly | 104 (0.3) | 104 (0.3) | 135 (0.4) |  |
| 1-3 days per week | 514 (1.5) | 493 (1.6) | 607 (1.7) |  |
| 4-6 days per week | 1,326 (4.0) | 1,209 (4.0) | 1,461 (4.0) |  |
| Daily | 31,319 (94.1) | 28,627 (94.0) | 34,245 (94.0) |  |
| Whole grains (%) |  |  |  |  |
| Never/rarely | 10,062 (30.3) | 8,913 (29.3) | 10,493 (28.8) | <0.001 |
| Monthly | 14,969 (45.0) | 13,845 (45.5) | 16,434 (45.1) |  |
| 1-3 days per week | 3,832 (11.5) | 3,303 (10.9) | 4,081 (11.2) |  |
| 4-6 days per week | 240 (0.7) | 211 (0.7) | 254 (0.7) |  |
| Daily | 4,174 (12.5) | 4,169 (13.7) | 5,195 (14.3) |  |
| Meats (%) |  |  |  |  |
| Never/rarely | 1,715 (5.2) | 1,690 (5.6) | 2,014 (5.5) | <0.001 |
| Monthly | 4,436 (13.3) | 4,236 (13.9) | 5,233 (14.4) |  |
| 1-3 days per week | 12,136 (36.5) | 11,115 (36.5) | 13,377 (36.7) |  |
| 4-6 days per week | 5,969 (17.9) | 5,263 (17.3) | 6,399 (17.6) |  |
| Daily | 9,021 (27.1) | 8,137 (26.7) | 9,434 (25.9) |  |
| Physical activity MET hours/day (S.D) | 21.9 (14.0) | 21.6 (14.0) | 21.8 (13.9) | 0.62 |
| Body mass index (S.D) | 23.5 (3.4) | 23.4 (3.4) | 23.4 (3.3) | 0.04 |
| Waist hip ratio (S.D) | 0.88 (0.1) | 0.88 (0.1) | 0.88 (0.1) | 0.53 |

^1^ P-values computed via ANOVA for continuous variables and chi-square test for categorical variables.

**Supplementary Table 7. Baseline characteristics by DM-GRS category in the SCHS^1^**

| **Characteristic** | **Low DM-GRS (N=4,764)** | **Middle DM-GRS**  **(N=5,233)** | **High DM-GRS**  **(N=6,175)** | **P-value** |
| --- | --- | --- | --- | --- |
| Age (S.D) | 54.3 (7.2) | 54.7 (7.2) | 54.5 (7.3) | 0.22 |
| Male (%) | 2,054 (43.1) | 2,341 (44.7) | 2,651 (42.9) | 0.12 |
| Smoking (%) |  |  |  | 0.92 |
| Never or occasional smoker | 3,471 (72.9) | 3,792 (72.5) | 4,514 (73.1) |  |
| Ex-smoker | 456 (9.6) | 521 (10.0) | 591 (9.6) |  |
| Current smoker: 1-12 cig/day | 333 (7.0) | 363 (6.9) | 404 (6.5) |  |
| Current smoker: 13-22 cig/day | 363 (7.6) | 383 (7.3) | 473 (7.7) |  |
| Current smoker: 23+ cig/day | 141 (3.0) | 174 (3.3) | 193 (3.1) |  |
| Alcohol (%) |  |  |  |  |
| Men：10-25 g/d；Women：5-15 g/d | 162 (3.4) | 173 (3.3) | 182 (3.0) | 0.35 |
| Vegetable-fruit-soy pattern (S.D) | 1.5 (1.1) | 1.5 (1.1) | 1.5 (1.1) | 0.63 |
| Meat-dim-sum pattern (S.D) | 1.5 (1.1) | 1.4 (1.1) | 1.4 (1.1) | 0.24 |
| Physical activity h/week (S.D) | 1.6 (4.1) | 1.8 (4.5) | 1.7 (4.4) | 0.36 |
| Body mass index (S.D) | 23.1 (3.4) | 23.0 (3.5) | 23.0 (3.4) | 0.23 |
|  |  |  |  |  |

^1^ P-values computed via ANOVA for continuous variables and chi-square test for categorical variables.

**Supplementary Table 8. Association between quintiles of BC-GRS, IR-GRS and Type 2 Diabates^1^**

|  |  | **Quintiles of score** | | | | | **P for trend** |
| --- | --- | --- | --- | --- | --- | --- | --- |
|  | **Continuous score**  **(Total)** | **Quintiles 1 (lowest)** | **Quintiles 2** | **Quintiles 3** | **Quintiles 4** | **Quintiles 5 (highest)** |  |
| **CKB** |  |  |  |  |  |  |  |
| BC-GRS |  |  |  |  |  |  |  |
| Number of type 2 diabetes | 3,383 | 458 | 647 | 451 | 889 | 938 |  |
| Mean(range) | 27.5 (14.0,40.0) | 23.0 (14.0, 24.9) | 25.6 (25.0, 26.9) | 27.1 (27.0, 27.9) | 28.5 (28.0, 29.9) | 31.3 (30.0, 40.0) |  |
| Age adjusted | 1.10 (1.08, 1.13) | 1.00 | 1.05 (0.93, 1.19) | 1.20 (1.05, 1.36) | 1.29 (1.16, 1.45) | 1.45 (1.30, 1.63) | <0.001 |
| Multivariate adjusted | 1.11 (1.09, 1.14) | 1.00 | 1.05 (0.93, 1.19) | 1.21 (1.06, 1.38) | 1.32 (1.18, 1.47) | 1.50 (1.34, 1.68) | <0.001 |
| IR-GRS |  |  |  |  |  |  |  |
| Number of type 2 diabetes | 3,383 | 494 | 706 | 965 | 789 | 429 |  |
| Mean(range) | 8.9 (3.0,12.0) | 6.6 (3.0, 7.9) | 8.0 (8.0, 8.9) | 9.0 (9.0, 9.0) | 10.0 (9.1, 10.0) | 11.2 (10.1, 12.0) |  |
| Age adjusted | 1.02 (0.99, 1.05) | 1.00 | 1.05 (0.94, 1.18) | 1.12 (1.01, 1.25) | 1.12 (1.00, 1.25) | 1.07 (0.94, 1.22) | 0.12 |
| Multivariate adjusted | 1.02 (0.99, 1.05) | 1.00 | 1.06 (0.94, 1.19) | 1.12 (1.01, 1.25) | 1.11 (0.99, 1.24) | 1.07 (0.94, 1.22) | 0.15 |
|  |  |  |  |  |  |  |  |
| **SCHS** |  |  |  |  |  |  |  |
| BC-GRS |  |  |  |  |  |  |  |
| Number of type 2 diabetes | 2,036 | 237 | 418 | 273 | 558 | 550 |  |
| Mean(range) | 23.2 (12.0, 34.0) | 19.0 (12.0, 20.9) | 21.6 (21.0, 22.7) | 23.0 (22.8, 23.9) | 24.5 (24.0, 25.9) | 27.1 (26.0, 34.0) |  |
| Age adjusted | 1.08 (1.07, 1.10) | 1.00 | 1.33 (1.13, 1.56) | 1.34 (1.13, 1.60) | 1.64 (1.41, 1.91) | 2.00 (1.72, 2.33) | <0.001 |
| Multivariate adjusted | 1.08 (1.07, 1.10) | 1.00 | 1.32 (1.13, 1.55) | 1.33 (1.12, 1.59) | 1.64 (1.41, 1.91) | 1.99 (1.71, 2.32) | <0.001 |
| IR-GRS |  |  |  |  |  |  |  |
| Number of type 2 diabetes | 2,036 | 91 | 306 | 673 | 676 | 290 |  |
| Mean (range) | 6.3 (1.0, 8.0) | 3.8 (1.0, 4.1) | 5.0 (5.0, 5.7) | 6.0 (6.0, 6.7) | 7.0 (7.0, 7.0) | 8.0 (7.1, 8.0) |  |
| Age adjusted | 1.02 (0.98, 1.06) | 1.00 | 1.04 (0.82, 1.32) | 1.11 (0.89, 1.38) | 1.06 (0.85, 1.32) | 1.15 (0.91, 1.45) | 0.32 |
| Multivariate adjusted | 1.02 (0.98, 1.07) | 1.00 | 1.05 (0.83, 1.32) | 1.11 (0.89, 1.39) | 1.07 (0.86, 1.33) | 1.15 (0.91, 1.46) | 0.29 |
|  |  |  |  |  |  |  |  |
| **Pooled** |  |  |  |  |  |  |  |
| BC-GRS |  |  |  |  |  |  |  |
| Number of type 2 diabetes | 5,419 | 695 | 1065 | 724 | 1447 | 1488 |  |
| Age adjusted |  | 1.00 | 1.15 (1.04, 1.26) | 1.26 (1.13, 1.40) | 1.41 (1.28, 1.54) | 1.58 (1.44, 1.73) | <0.001 |
| Multivariate adjusted |  | 1.00 | 1.15 (1.05, 1.27) | 1.26 (1.13, 1.40) | 1.44 (1.31, 1.58) | 1.69 (1.54, 1.85) | <0.001 |
| IR-GRS |  |  |  |  |  |  |  |
| Number of type 2 diabetes | 5,419 | 585 | 1012 | 1638 | 1465 | 719 |  |
| Age adjusted |  | 1.00 | 1.16 (1.05, 1.29) | 1.34 (1.22, 1.48) | 1.37 (1.25, 1.51) | 1.29 (1.16, 1.44) | <0.001 |
| Multivariate adjusted |  | 1.00 | 1.06 (0.95, 1.17) | 1.12 (1.02, 1.23) | 1.09 (0.99, 1.20) | 1.11 (1.00, 1.24) | 0.06 |

^1^ Data are HR (95%CI) unless otherwise indicated. Adjusted for sex, age, region code, data sources and family history of diabetes in the CKB cohort and adjusted for sex, age, education, father dialect and years of interview in the SCHS cohort. The participants were combined by quintile of lifestyle score or genetic risk score among each cohort and model was adjusted for sex, age, region and data sources. Those in the lowest quintile of genetic risk serve as the reference group.

**Supplementary Table 9. Interplay between single SNP and lifestyle score in the CKB^1^**

| **SNP** | **β-coefficient** | **S.E** | **P for interaction** |
| --- | --- | --- | --- |
| rs340874 | -0.014 | 0.008 | 0.085 |
| rs7578597 | -0.022 | 0.046 | 0.637 |
| rs11708067 | 0.061 | 0.079 | 0.441 |
| rs1470579 | 0.004 | 0.009 | 0.624 |
| rs16861329 | 0.019 | 0.010 | 0.053 |
| rs6815464 | 0.001 | 0.008 | 0.880 |
| rs7754840 | -0.013 | 0.008 | 0.098 |
| rs4607517 | 0.011 | 0.009 | 0.251 |
| rs6467136 | 0.001 | 0.011 | 0.974 |
| rs13266634 | 0.001 | 0.007 | 0.998 |
| rs7041847 | -0.005 | 0.008 | 0.502 |
| rs10811661 | -0.001 | 0.008 | 0.891 |
| rs1111875 | -0.004 | 0.009 | 0.675 |
| rs7901695 | -0.035 | 0.021 | 0.102 |
| rs4752781 | -0.004 | 0.011 | 0.708 |
| rs2237892 | -0.003 | 0.008 | 0.742 |
| rs5215 | -0.008 | 0.008 | 0.313 |
| rs1552224 | -0.025 | 0.014 | 0.088 |
| rs10830963 | -0.011 | 0.008 | 0.154 |
| rs1359790 | 0.001 | 0.009 | 0.989 |
| rs7172432 | -0.012 | 0.008 | 0.147 |
| rs2028299 | -0.001 | 0.010 | 0.991 |
| rs8042680 | -0.010 | 0.044 | 0.810 |
| rs4430796 | 0.004 | 0.009 | 0.681 |
| rs6017317 | 0.011 | 0.009 | 0.220 |
| rs780094 | 0.013 | 0.008 | 0.102 |
| rs7593730 | 0.007 | 0.011 | 0.493 |
| rs3923113 | -0.008 | 0.011 | 0.482 |
| rs2943641 | 0.005 | 0.015 | 0.733 |
| rs831571 | 0.002 | 0.008 | 0.807 |
| rs972283 | -0.005 | 0.009 | 0.586 |
| rs10923931 | 0.034 | 0.022 | 0.115 |
| rs243021 | -0.003 | 0.008 | 0.764 |
| rs6780569 | -0.013 | 0.010 | 0.194 |
| rs4607103 | -0.005 | 0.008 | 0.569 |
| rs4457053 | -0.004 | 0.017 | 0.797 |
| rs9470794 | -0.005 | 0.008 | 0.587 |
| rs864745 | 0.003 | 0.009 | 0.711 |
| rs896854 | -0.003 | 0.008 | 0.738 |
| rs17584499 | 0.018 | 0.013 | 0.175 |
| rs13292136 | -0.014 | 0.013 | 0.290 |
| rs10906115 | -0.006 | 0.008 | 0.465 |
| rs1802295 | 0.021 | 0.012 | 0.095 |
| rs7961581 | -0.007 | 0.009 | 0.459 |
| rs7178572 | 0.002 | 0.008 | 0.787 |
| rs11634397 | 0.023 | 0.014 | 0.090 |
| rs9939609 | -0.025 | 0.011 | 0.030 |
| rs4523957 | 0.001 | 0.009 | 0.879 |
| rs5945326 | 0.005 | 0.007 | 0.437 |

^1^ Model was adjusted for sex, age, region code, data sources and family history of diabetes in the CKB cohort. We used likelihood ratio test to compare models with and without cross-product term to test the interaction.

**Supplementary Table 10. Interplay between single SNP and lifestyle score in the SCHS^1^**

| **SNP** | **β-coefficient** | **S.E** | **P for interaction** |
| --- | --- | --- | --- |
| rs340874 | -0.003 | 0.013 | 0.798 |
| rs7578597 | -0.037 | 0.081 | 0.648 |
| rs11708067 | 0.032 | 0.153 | 0.835 |
| rs1470579 | 0.003 | 0.015 | 0.866 |
| rs16861329 | 0.001 | 0.016 | 0.971 |
| rs6815464 | -0.015 | 0.013 | 0.241 |
| rs7754840 | -0.009 | 0.013 | 0.505 |
| rs4607517 | 0.001 | 0.017 | 0.928 |
| rs6467136 | -0.004 | 0.017 | 0.809 |
| rs13266634 | -0.001 | 0.013 | 0.916 |
| rs7041847 | -0.010 | 0.013 | 0.460 |
| rs10811661 | -0.014 | 0.013 | 0.313 |
| rs7901695 | 0.051 | 0.038 | 0.172 |
| rs2237892 | -0.016 | 0.014 | 0.269 |
| rs5215 | -0.007 | 0.013 | 0.583 |
| rs1552224 | 0.012 | 0.027 | 0.646 |
| rs10830963 | 0.013 | 0.013 | 0.322 |
| rs1359790 | -0.010 | 0.015 | 0.482 |
| rs7172432 | -0.011 | 0.014 | 0.443 |
| rs2028299 | 0.006 | 0.016 | 0.706 |
| rs4430796 | 0.014 | 0.015 | 0.338 |
| rs6017317 | -0.006 | 0.013 | 0.644 |
| rs780094 | -0.022 | 0.013 | 0.094 |
| rs7593730 | 0.011 | 0.018 | 0.539 |
| rs3923113 | 0.010 | 0.020 | 0.612 |
| rs2943641 | -0.029 | 0.026 | 0.271 |
| rs243021 | -0.026 | 0.014 | 0.063 |
| rs4607103 | 0.025 | 0.014 | 0.065 |
| rs4457053 | 0.007 | 0.027 | 0.795 |
| rs17584499 | 0.027 | 0.020 | 0.175 |
| rs13292136 | -0.008 | 0.023 | 0.721 |
| rs10906115 | -0.022 | 0.013 | 0.097 |
| rs1802295 | -0.010 | 0.020 | 0.607 |
| rs7961581 | -0.005 | 0.015 | 0.740 |
| rs7178572 | -0.005 | 0.013 | 0.694 |
| rs11634397 | -0.005 | 0.024 | 0.850 |
| rs4523957 | -0.010 | 0.013 | 0.457 |

^1^ Model was adjusted for sex, age, education, father dialect and years of interview in the SCHS cohort. We used likelihood ratio test to compare models with and without cross-product term to test the interaction.

**Supplementary Table 11. Interplay between single character and genetic risk in the CKB^1^**

| **Character** | **β-coefficient** | **S.E** | **P for interaction** |
| --- | --- | --- | --- |
| Age | -0.001 | 0.001 | 0.381 |
| Sex | -0.006 | 0.009 | 0.486 |
| Alcohol | 0.019 | 0.012 | 0.122 |
| Smoking | 0.004 | 0.010 | 0.718 |
| Meat | -0.002 | 0.004 | 0.609 |
| Vegetable | -0.007 | 0.015 | 0.640 |
| Fruits | 0.004 | 0.004 | 0.303 |
| Whole grain | -0.003 | 0.004 | 0.505 |
| Physical activity | 0.001 | 0.001 | 0.599 |
| BMI | 0.001 | 0.001 | 0.291 |
| Waist to hip ratio | -0.404 | 0.025 | <10^-8^ |

^1^ Model was adjusted for sex, age, region code, diet (fruits, vegetables, whole grain and whole grain), alcohol (nondrinker and current drinker), smoking (nonsmoker and current smoker), physical activity, BMI, data sources and family history of diabetes in the CKB cohort. We used likelihood ratio test to compare models with and without cross-product term to test the interaction.

**Supplementary Table 12. Interplay between single character and DM-GRS in the SCHS^1^**

| **Character** | **β-coefficient** | **S.E** | **P for interaction** |
| --- | --- | --- | --- |
| Age | -0.001 | 0.001 | 0.129 |
| Sex | 0.016 | 0.014 | 0.233 |
| Alcohol | -0.015 | 0.017 | 0.355 |
| Smoking | -0.05 | 0.018 | 0.004 |
| Vegetable-fruit-soy pattern | -0.008 | 0.006 | 0.268 |
| Meat-dim-sum pattern | 0.003 | 0.006 | 0.665 |
| Physical activity | -0.001 | 0.002 | 0.426 |
| BMI | -0.005 | 0.001 | 0.001 |

^1^ Model was adjusted for sex, age, father dialect, years of interview, vegetable-fruit-soy pattern, meat-dim-sum pattern, alcohol (nondrinker and current drinker), smoking (nonsmoker and current smoker), physical activity and BMI in the SCHS cohort.

**Supplementary Table 13 Subgroup analyses stratified by lifestyle factors in the CKB^1^**

|  | **Healthful lifestyle** | |  | **Unhealthful lifestyle** | | **P for Heterogeneity** |
| --- | --- | --- | --- | --- | --- | --- |
|  | **Low GRS** | **High GRS** |  | **Low GRS** | **High GRS** |  |
| **Age (N)** |  |  |  |  |  | 0.78 |
| **< 45 (32,052)** | 1.00 | 1.53 (0.95, 2.45) |  | 3.38 (2.14, 5.34) | 5.02 (3.26, 7.73) |  |
| **45-59 (43,211)** | 1.00 | 1.47 (1.05, 2.06) |  | 2.81 (2.08, 3.79) | 4.03 (3.02, 5.39) |  |
| **> 59 (24,912)** | 1.00 | 2.36 (1.32, 4.21) |  | 2.93 (1.75, 4.91) | 4.56 (2.74, 7.59) |  |
| **Sex** |  |  |  |  |  | 0.06 |
| **Man (42,127)** | 1.00 | 1.10 (0.63, 1.89) |  | 1.84 (1.20, 2.84) | 2.85 (1.86, 4.36) |  |
| **Woman (58,048)** | 1.00 | 1.80 (1.36, 2.38) |  | 3.23 (2.48, 4.20) | 4.64 (3.60, 5.98) |  |
| **Alcohol** |  |  |  |  |  | 0.24 |
| **Healthful (3,277)** | 1.00 | 1.70 (0.42, 6.92) |  | 1.78 (0.49, 6.50) | 3.10 (0.90, 10.7) |  |
| **Unhealthful (96,898)** | 1.00 | 1.62 (1.26, 2.08) |  | 2.96 (2.36, 3.71) | 4.36 (3.51, 5.43) |  |
| **Smoking** |  |  |  |  |  | 0.47 |
| **Never or occasional smoker (66,344)** | 1.00 | 1.65 (1.28, 2.12) |  | 3.09 (2.42, 3.94) | 4.50 (3.57, 5.67) |  |
| **Ex-smoker (5,854)** | 1.00 | 0.43 (0.03, 4.73) |  | 4.42 (1.06, 18.5) | 7.49 (1.82, 30.9) |  |
| **Current smoker: 1-9 cig/day (5,737)** | 1.00 | 3.67 (0.43, 31.5) |  | 4.70 (0.62, 35.6) | 6.35 (0.85, 47.5) |  |
| **Current smoker:**  **10-19 cig/day (7,735)** | 1.00 | 1.27 (0.21, 7.65) |  | 1.35 (0.32, 5.76) | 1.54 (0.36, 6.52) |  |
| **Current smoker: ≥20 cig/day (14,505)** | 1.00 | 0.67 (0.08, 5.50) |  | 1.54 (0.21, 11.3) | 2.59 (0.35, 19.0) |  |
| **Meat** |  |  |  |  |  | 0.07 |
| **Not Daily (73,583)** | 1.00 | 1.56 (1.20, 2.01) |  | 2.77 (2.18, 3.53) | 4.16 (3.30, 5.23) |  |
| **Daily (26,592)** | 1.00 | 2.47 (1.04, 5.87) |  | 5.50 (2.57, 11.8) | 8.00 (3.76, 17.0) |  |
| **Fruits** |  |  |  |  |  | 0.32 |
| **Daily (16,373)** | 1.00 | 1.30 (0.84, 2.01) |  | 3.00 (1.92, 4.70) | 3.65 (2.37, 5.63) |  |
| **Not Daily (83,802)** | 1.00 | 1.79 (1.32, 2.42) |  | 3.03 (2.32, 3.96) | 4.61 (3.55, 5.99) |  |
| **Vegetable** |  |  |  |  |  | 0.22 |
| **Daily (94,191)** | 1.00 | 1.65 (1.28, 2.11) |  | 3.04 (2.43, 3.81) | 4.55 (3.65, 5.66) |  |
| **Not Daily (5,984)** | 1.00 | 1.12 (0.22, 5.62) |  | 1.69 (0.49, 5.78) | 2.29 (0.68, 7.66) |  |
| **Whole grain** |  |  |  |  |  | 0.65 |
| **Daily (13,538)** | 1.00 | 1.25 (0.75, 2.10) |  | 0.38 (0.05, 2.86) | 5.50 (2.85, 10.6) |  |
| **Not Daily (86,637)** | 1.00 | 1.76 (1.33, 2.33) |  | 3.14 (2.45, 4.03) | 4.59 (3.61, 5.85) |  |
| **Physical activity** |  |  |  |  |  | 0.08 |
| **Highest quintile (20,039)** | 1.00 | 2.01 (1.32, 3.04) |  | 4.31 (2.74, 6.79) | 5.29 (3.42, 8.19) |  |
| **Second quintile (20,035)** | 1.00 | 1.31 (0.85, 2.04) |  | 1.99 (1.28, 3.08) | 3.37 (2.26, 5.04) |  |
| **Third quintile (20,193)** | 1.00 | 1.31 (0.75, 2.26) |  | 2.94 (1.82, 4.77) | 4.29 (2.69, 6.85) |  |
| **Fourth quintile (19,874)** | 1.00 | 1.79 (0.81, 3.95) |  | 4.79 (2.39, 9.59) | 7.27 (3.66, 14.5) |  |
| **Lowest quintile (20,034)** | 1.00 | 4.57 (0.55, 38.0) |  | 12.6 (1.75, 90.3) | 18.8 (2.62, 135.2) |  |
| **BMI** |  |  |  |  |  | 0.97 |
| **<23.9 kg/m^2^ (58,867)** | 1.00 | 1.74 (1.26, 2.41) |  | 1.77 (1.27, 2.45) | 2.84 (2.09, 3.85) |  |
| **24.0-27.9 kg/m^2^ (31,677)** | 1.00 | 1.80 (1.15, 2.80) |  | 1.73 (1.16, 2.57) | 2.74 (1.86, 4.03) |  |
| **≥28.0 kg/m^2^ (9,631)** | 1.00 | 0.77 (0.35, 1.70) |  | 0.86 (0.48, 1.54) | 2.56 (0.68, 5.25) |  |
| **Waist-to-hip ratio** |  |  |  |  |  | 0.09 |
| **Low (43,054)** | 1.00 | 2.05 (1.43, 2.92) |  | 1.87 (1.25, 2.81) | 2.95 (2.04, 4.28) |  |
| **Middle (27,996)** | 1.00 | 1.35 (0.84, 2.16) |  | 1.36 (0.89, 2.07) | 2.03 (1.36, 3.05) |  |
| **High (29,125)** | 1.00 | 1.13 (0.68, 1.90) |  | 1.12 (0.73, 1.70) | 1.68 (1.11, 2.54) |  |

^1^ Model was adjusted for age, sex, region and family history of diabetes in the CKB.

**Supplementary Table 14 Subgroup analyses stratified by lifestyle factors in the SCHS^1^**

|  | **Healthful lifestyle** | |  | **Unhealthful lifestyle** | | **P for Heterogeneity** |
| --- | --- | --- | --- | --- | --- | --- |
|  | **Low GRS** | **High GRS** |  | **Low GRS** | **High GRS** |  |
| **Age (N)** |  |  |  |  |  | 0.04 |
| **45-59 (12,171)** | 1.00 | 1.93 (1.44, 2.58) |  | 2.54 (1.90, 3.38) | 4.18 (3.19, 5.47) |  |
| **> 59 (4,001)** | 1.00 | 1.47 (0.94, 2.30) |  | 1.50 (0.94, 2.37) | 2.23 (1.46, 3.41) |  |
| **Sex** |  |  |  |  |  | 0.79 |
| **Man (7,046)** | 1.00 | 2.16 (1.36, 3.42) |  | 2.60 (1.70, 4.00) | 3.61 (2.39, 5.47) |  |
| **Woman (9,126)** | 1.00 | 1.65 (1.24, 2.20) |  | 1.95 (1.43, 2.66) | 3.86 (2.94, 5.09) |  |
| **Alcohol** |  |  |  |  |  | 0.92 |
| **Healthful (517)** | 1.00 | 2.84 (0.29, 27.5) |  | 4.92 (0.64, 37.5) | 4.27 (0.55, 33.0) |  |
| **Unhealthful (15,655)** | 1.00 | 1.78 (1.39, 2.27) |  | 2.13 (1.67, 2.72) | 3.56 (2.84, 4.46) |  |
| **Smoking** |  |  |  |  |  | 0.12 |
| **Never or occasional smoker (11,777)** | 1.00 | 1.82 (1.41, 2.35) |  | 2.03 (1.52, 2.71) | 4.17 (3.25, 5.36) |  |
| **Ex-smoker (1,568)** | 1.00 | 1.33 (0.55, 3.21) |  | 1.94 (0.90, 4.19) | 2.36 (1.12, 4.97) |  |
| **Current smoker (2,827)** | 1.00 | 2.70 (0.28, 26.0) |  | 4.70 (0.65, 33.7) | 6.24 (0.87, 44.6) |  |
| **MDS** |  |  |  |  |  | 0.71 |
| **Lowest quartile (3,826)** | 1.00 | 1.83 (1.27, 2.63) |  | 2.75 (1.63, 4.66) | 3.26 (2.03, 5.25) |  |
| **Second quartile (4,066)** | 1.00 | 2.00 (1.27, 3.15) |  | 2.96 (1.88, 4.68) | 3.80 (2.45, 5.90) |  |
| **Third quartile (4,095)** | 1.00 | 1.18 (0.68, 2.05) |  | 1.44 (0.86, 2.39) | 3.20 (2.00, 5.11) |  |
| **Highest quartile (4,185)** | 1.00 | 2.89 (1.09, 7.71) |  | 3.18 (1.29, 7.84) | 5.05 (2.07, 12.3) |  |
| **VFS** |  |  |  |  |  | 0.73 |
| **Highest quartile (3,605)** | 1.00 | 1.65 (0.56, 4.83) |  | 1.83 (0.74, 4.52) | 2.92 (1.20, 7.13) |  |
| **Third quartile (4,064)** | 1.00 | 1.51 (0.80, 2.85) |  | 2.19 (1.25, 3.86) | 4.56 (2.67, 7.77) |  |
| **Second quartile (4,252)** | 1.00 | 2.04 (1.33, 3.14) |  | 2.93 (1.85, 4.64) | 4.07 (2.67, 6.22) |  |
| **Lowest quartile (4,251)** | 1.00 | 1.71 (1.21, 2.44) |  | 2.62 (1.65, 4.16) | 3.23 (2.12, 4.91) |  |
| **Physical activity** |  |  |  |  |  | 0.34 |
| **4+ h/wk (2,264)** | 1.00 | 1.61 (1.00, 2.58) |  | 1.91 (0.99, 3.66) | 2.43 (1.39, 4.28) |  |
| **0.5-<4 h/wk (3,510)** | 1.00 | 1.85 (1.17, 2.92) |  | 2.78 (1.68, 4.61) | 3.64 (2.29, 5.78) |  |
| **<0.5 h/wk (10,398)** | 1.00 | 1.87 (1.30, 2.69) |  | 2.14 (1.52, 3.00) | 3.66 (2.65, 5.06) |  |
| **BMI** |  |  |  |  |  | 0.91 |
| **<25.0 kg/m^2^ (10,564)** | 1.00 | 1.85 (1.40, 2.43) |  | 1.43 (1.02, 2.01) | 2.56 (1.92, 3.42) |  |
| **25-27.4 kg/m^2^ (2,225)** | 1.00 | 1.46 (0.61, 3.49) |  | 1.38 (0.66, 2.89) | 2.81 (1.37, 5.74) |  |
| **≥27.5 kg/m^2^ (3,383)** | 1.00 | 1.68 (0.88, 3.19) |  | 2.25 (1.29, 3.93) | 2.94 (1.70, 5.07) |  |

^1^ Model was adjusted for sex, age, father dialect, years of interview in the SCHS.

**Supplementary Figure 1. Flow chart of the analyses**

**Supplementary Figure 2. Distribution of lifestyle and genetic risk score in the CKB cohort**

Abbreviate: DM-GRS, Diabetes Genetic Risk Score; BC-GRS, Beta Cell Function Genetic Risk Score; IR-GRS, Insulin Resistance Genetic Risk Score.

**Supplementary Figure 3. Distribution of lifestyle and genetic risk score in the SCHS cohort**

Abbreviate: DM-GRS, Diabetes Genetic Risk Score; BC-GRS, Beta Cell Function Genetic Risk Score; IR-GRS, Insulin Resistance Genetic Risk Score.

**Supplementary Figure 4 Association between lifestyle score, DM-GRS, BC-GRS, IR-GRS and type 2 diabetes in the CKB cohort**

**a. Unadjusted family history of diabetes**


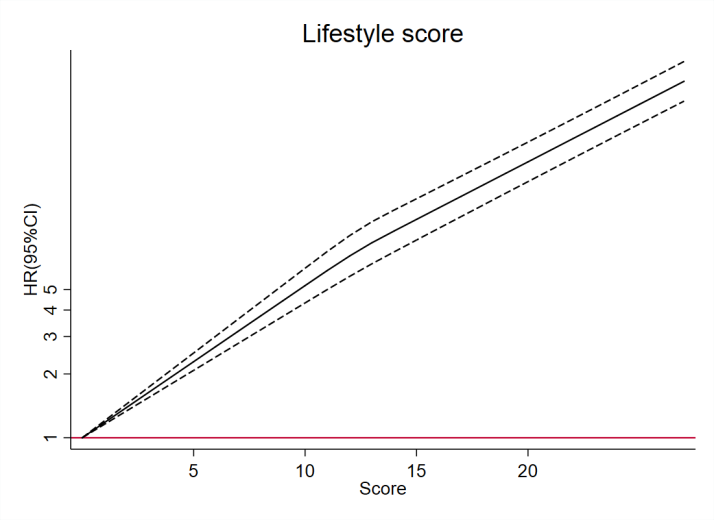

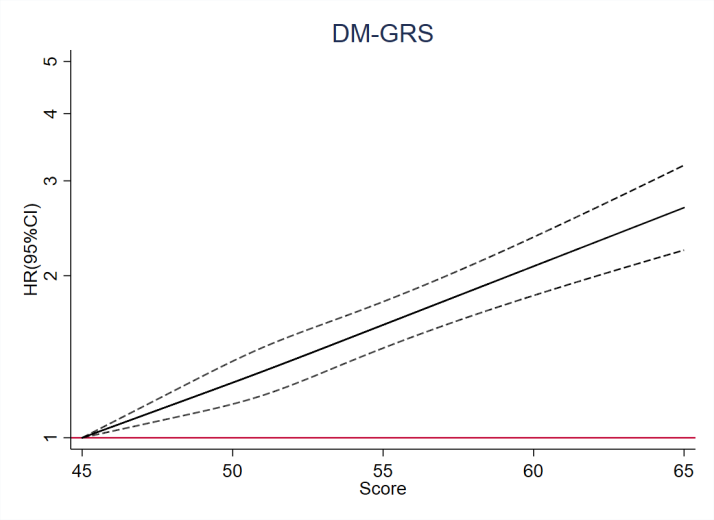


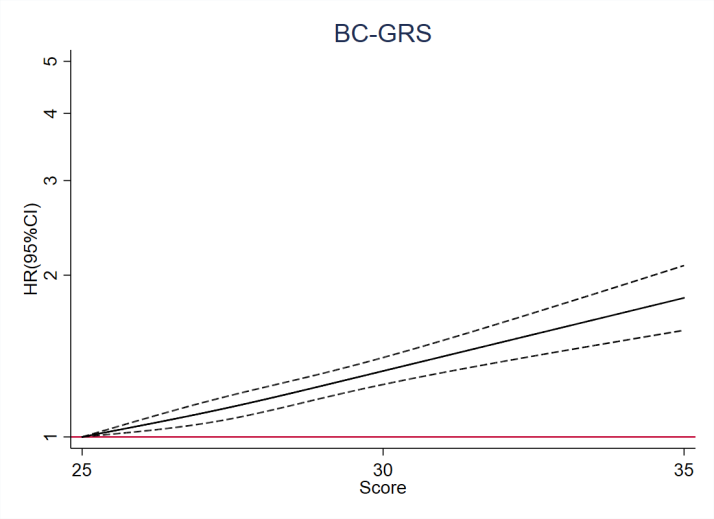

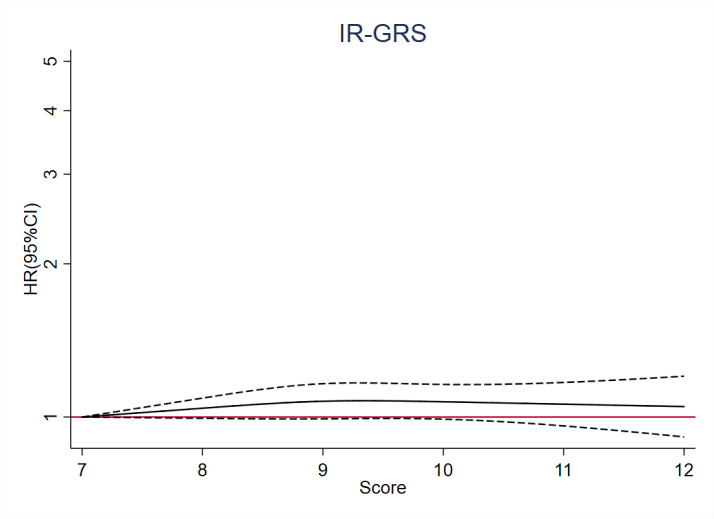


Model was fitted with restricted cubic splines and adjusted for age, sex and region. The solid line was hazard ratio; and the dash line was 95% confidence interval.

**b. Adjusted family history of diabetes**


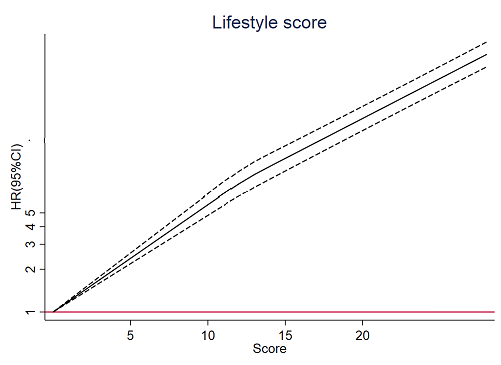

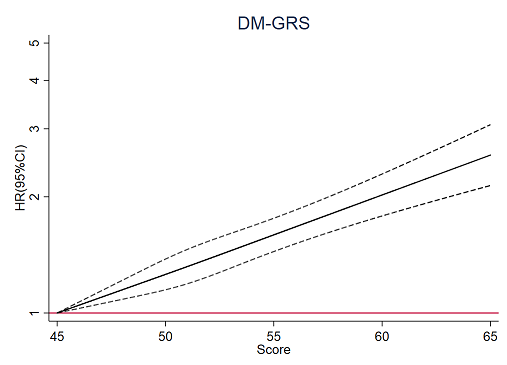


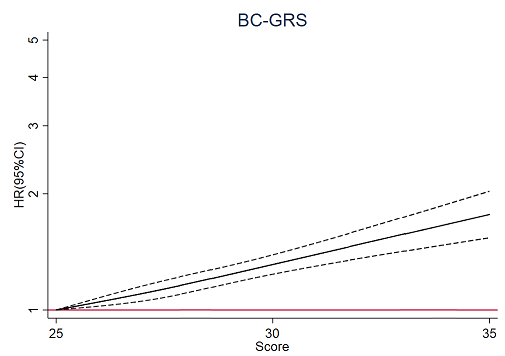

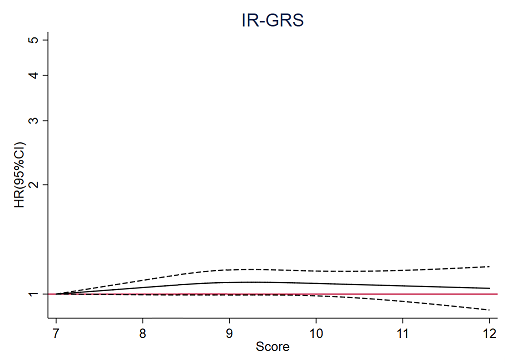


Model was fitted with restricted cubic splines and adjusted for age, sex, region and family history of diabetes. The solid line was hazard ratio; and the dash line was 95% confidence interval.

**Supplementary Figure 5. Association between lifestyle score, DM-GRS, BC-GRS, IR-GRS and type 2 diabetes in the SCHS cohort**


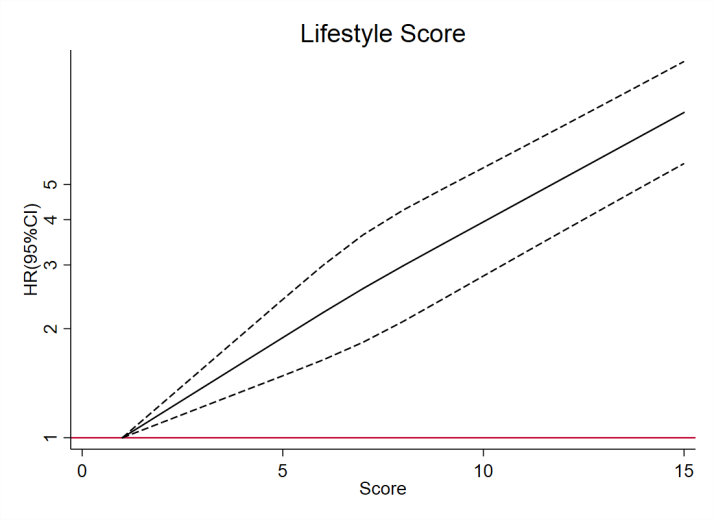

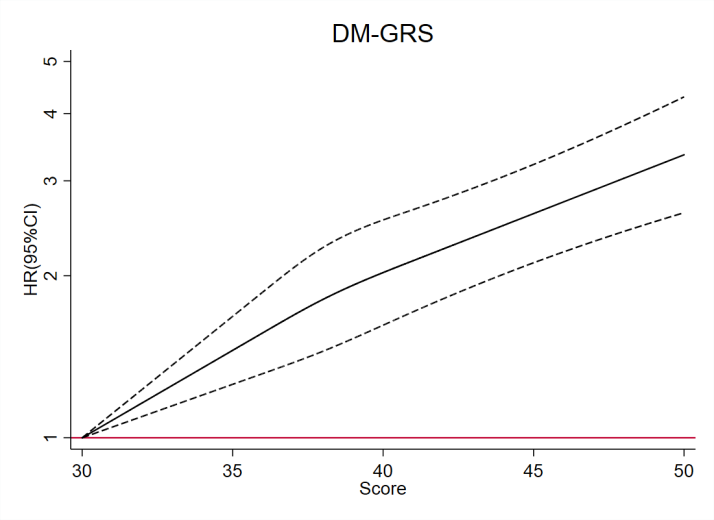


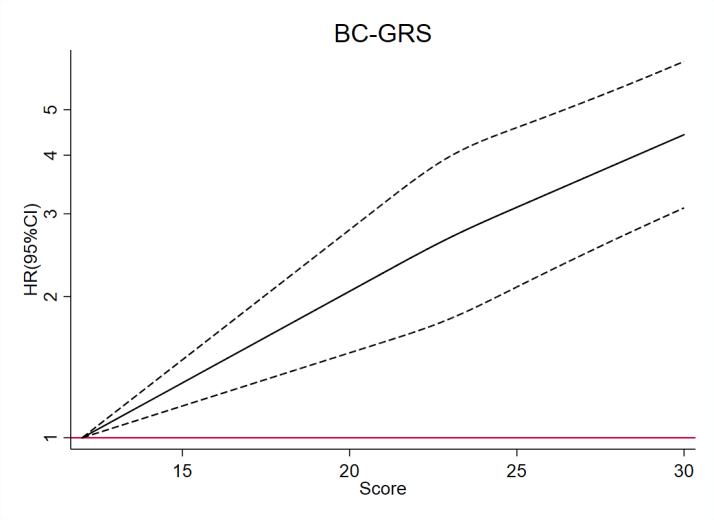

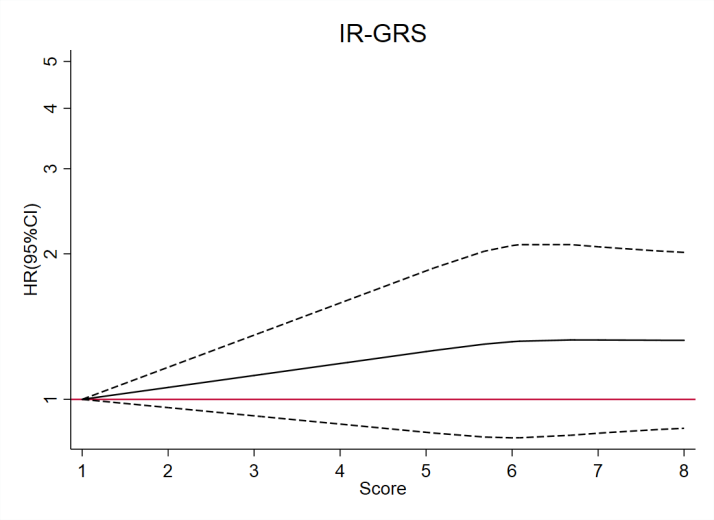


Model was fitted with restricted cubic splines and adjusted for sex, age, education, father dialect and years of interview. The solid line was hazard ratio; and the dash line was 95% confidence interval.

**Supplementary Figure 6. Adjusted type 2 diabetes events rates in the pooled cohort, according to DM-GRS, BC-GRS, IR-GRS and lifestyle score**


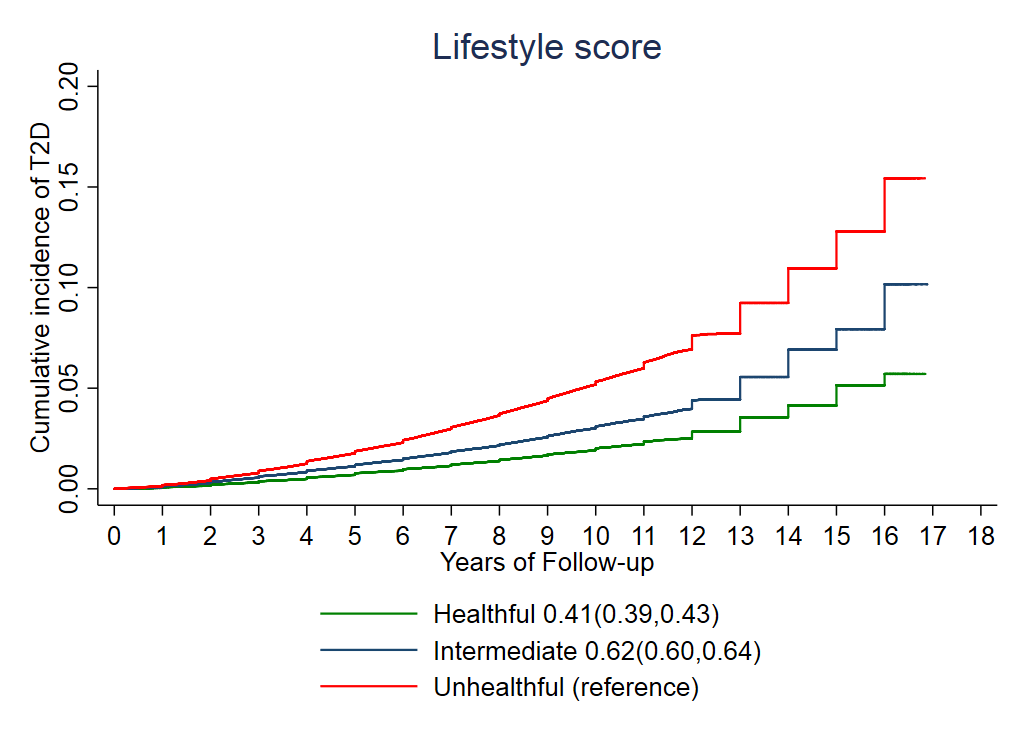

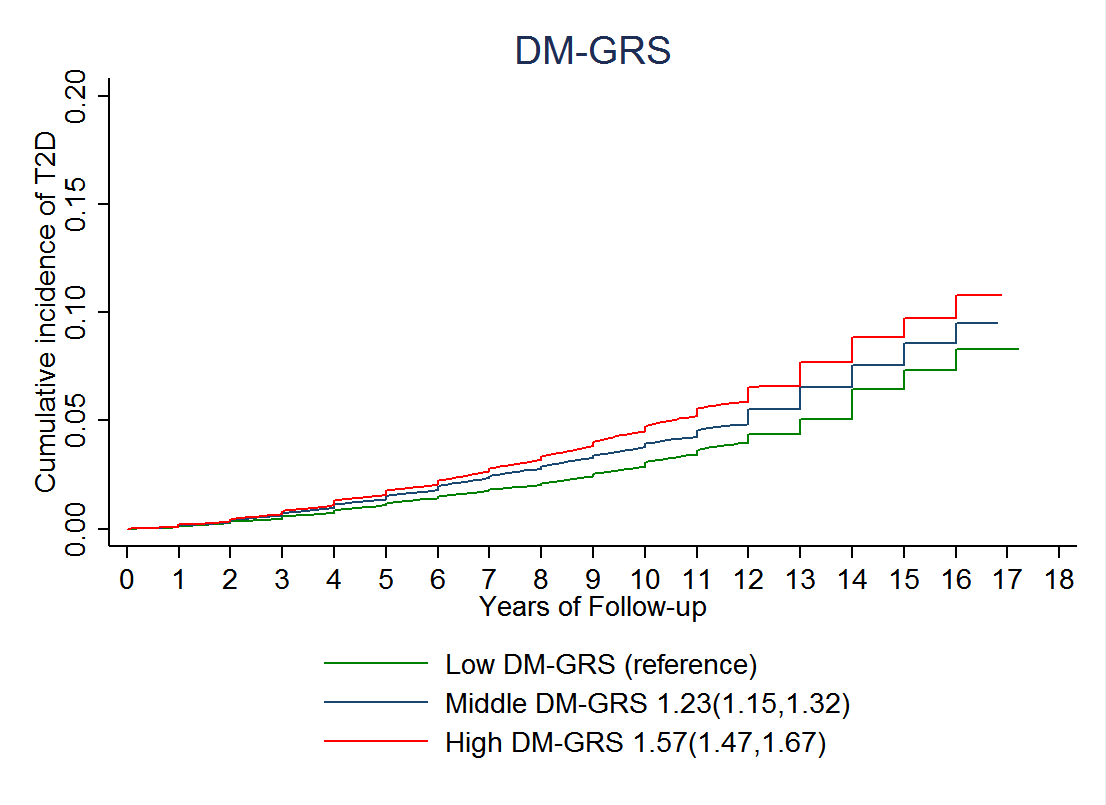


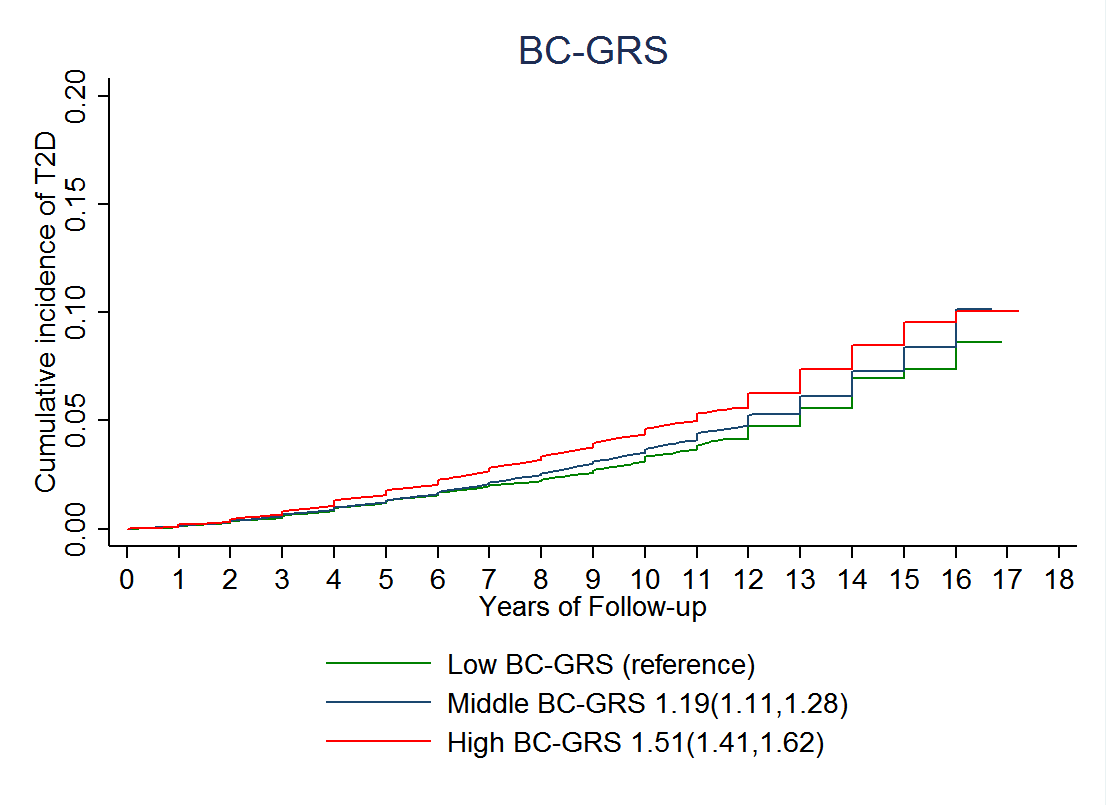

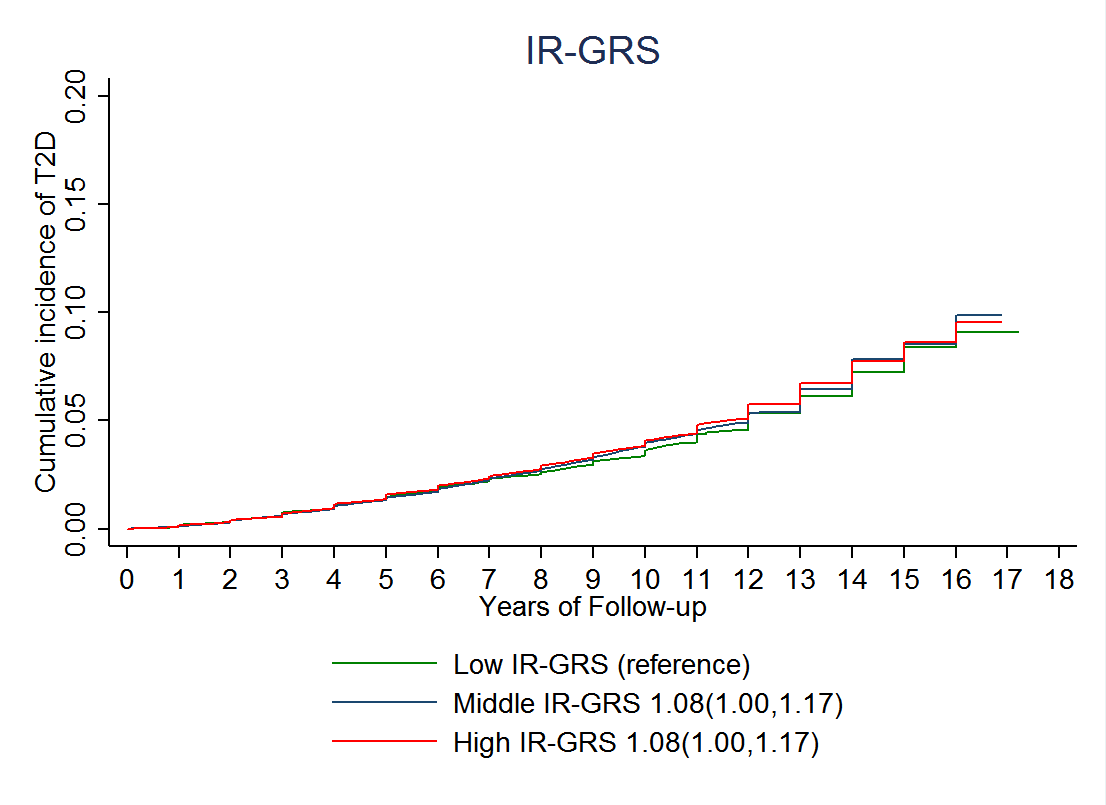


Shown are the adjusted rates of type 2 diabetes events in the pooled cohort, according to the DM-GRS, BC-GRS, IR-GRS and lifestyle score of participants. The 95% confidence intervals for the hazard ratios are provided in parentheses. Cox regression models were adjusted for age, sex, region and data source, which was performed to cohort-specific population averages for each covariate. All of P for trend was <0.001, except IR-GRS (P for trend=0.11).

**Supplementary Figure 7. Adjusted type 2 diabetes events rates, according to DM-GRS, BC-GRS, IR-GRS and lifestyle score in the CKB cohort**


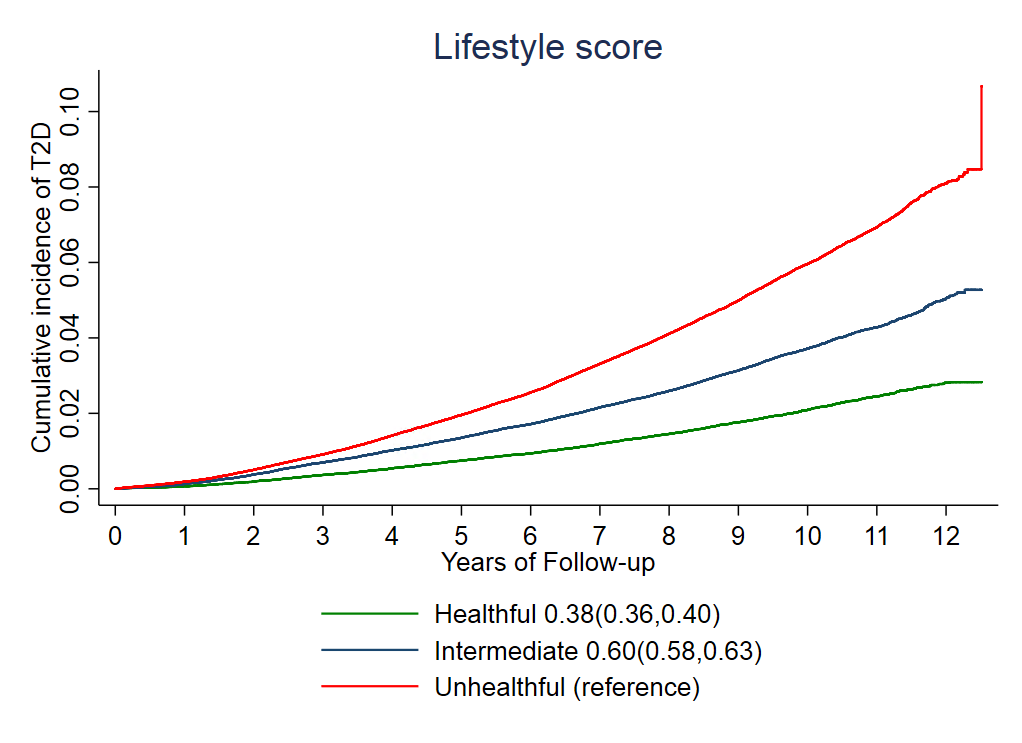

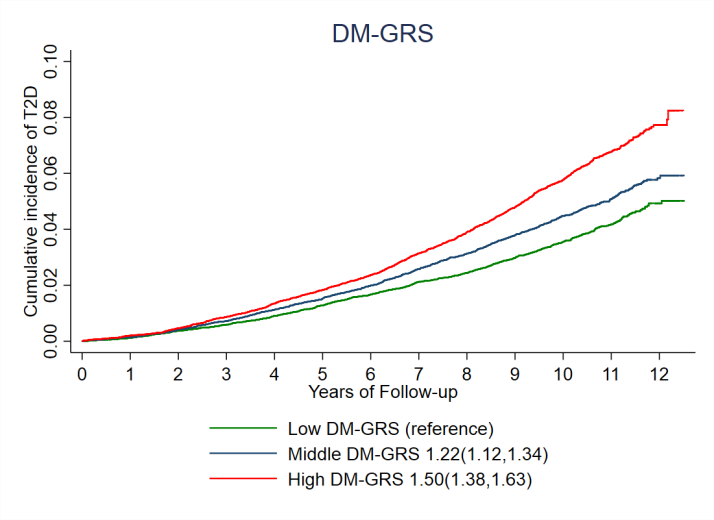


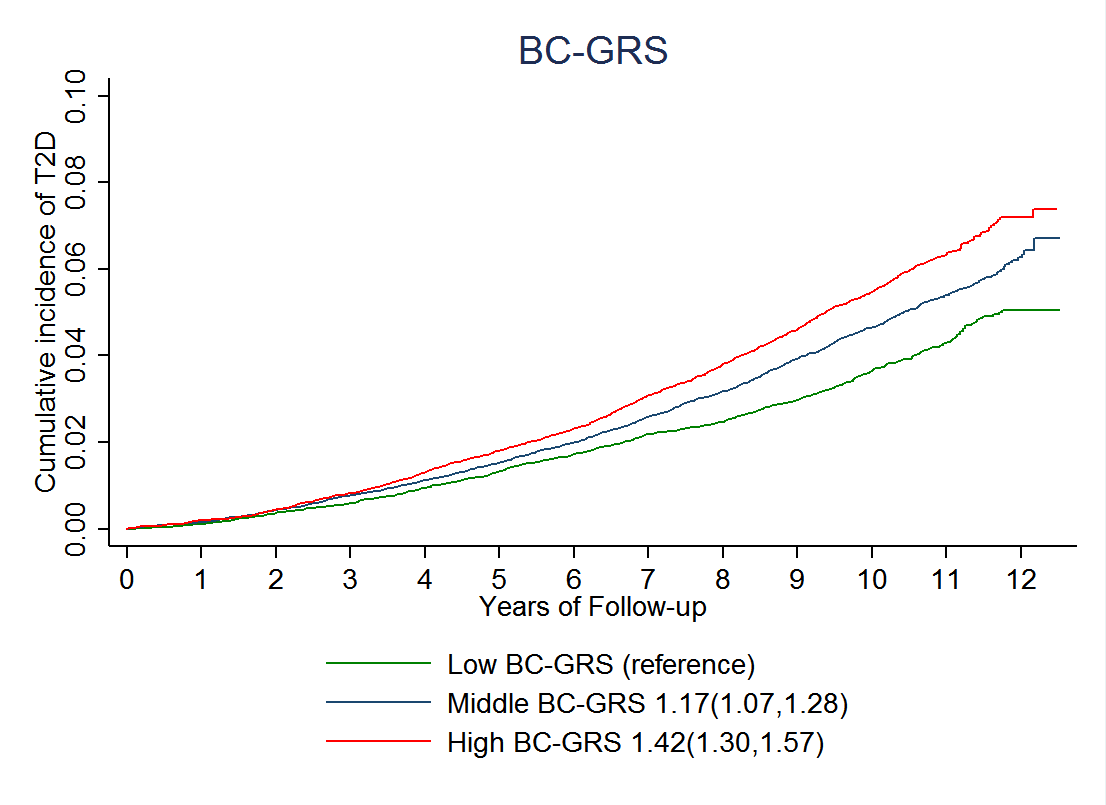

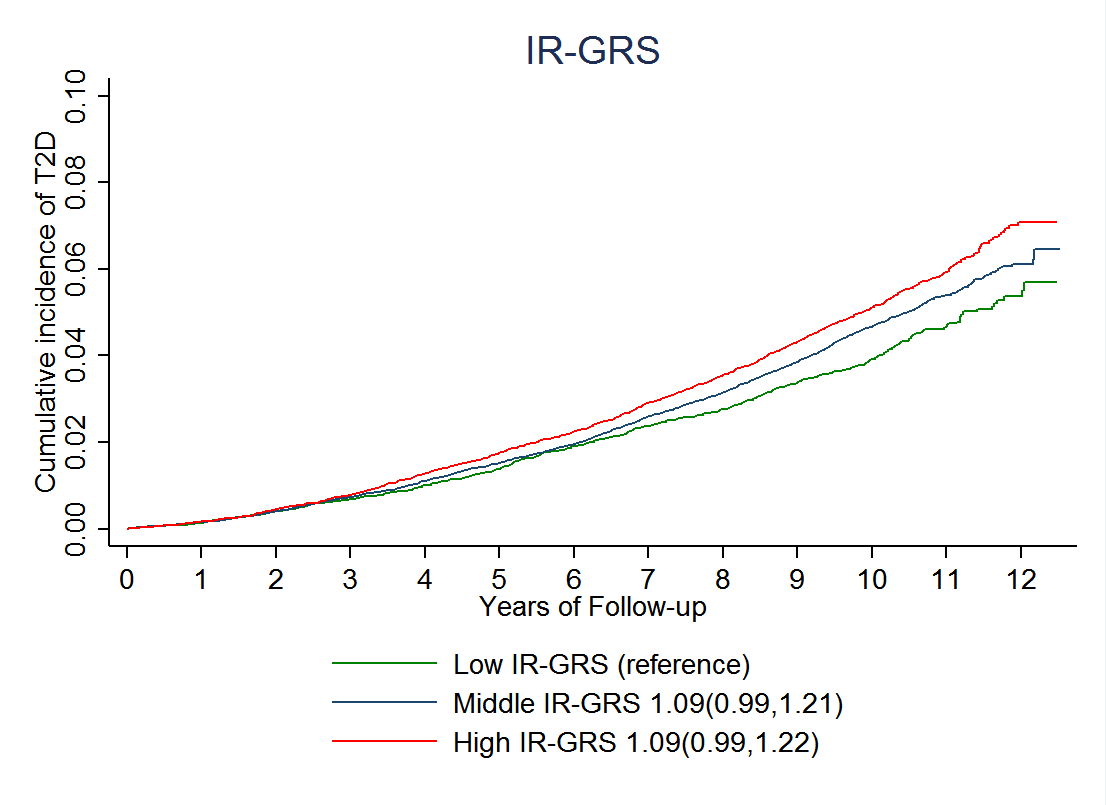


Shown are the adjusted rates of type 2 diabetes events, according to the DM-GRS, BC-GRS, IR-GRS and lifestyle score of participants. The 95% confidence intervals for the hazard ratios are provided in parentheses. Cox regression models were adjusted for age, sex, region, SNP data source, family history of diabetes, which was performed to cohort-specific population averages for each covariate. All of P for trend was <0.001, except IR-GRS (P for trend=0.16).

**Supplementary Figure 8. Adjusted type 2 diabetes events rates, according to DM-GRS, BC-GRS, IR-GRS and lifestyle score in the SCHS cohort**


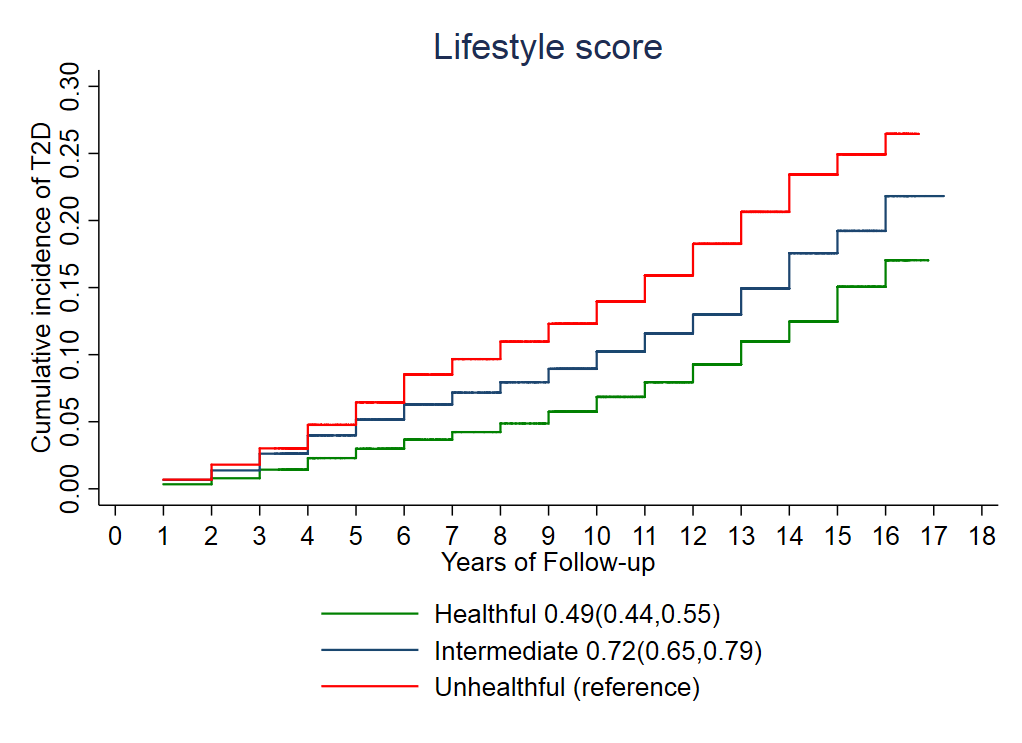

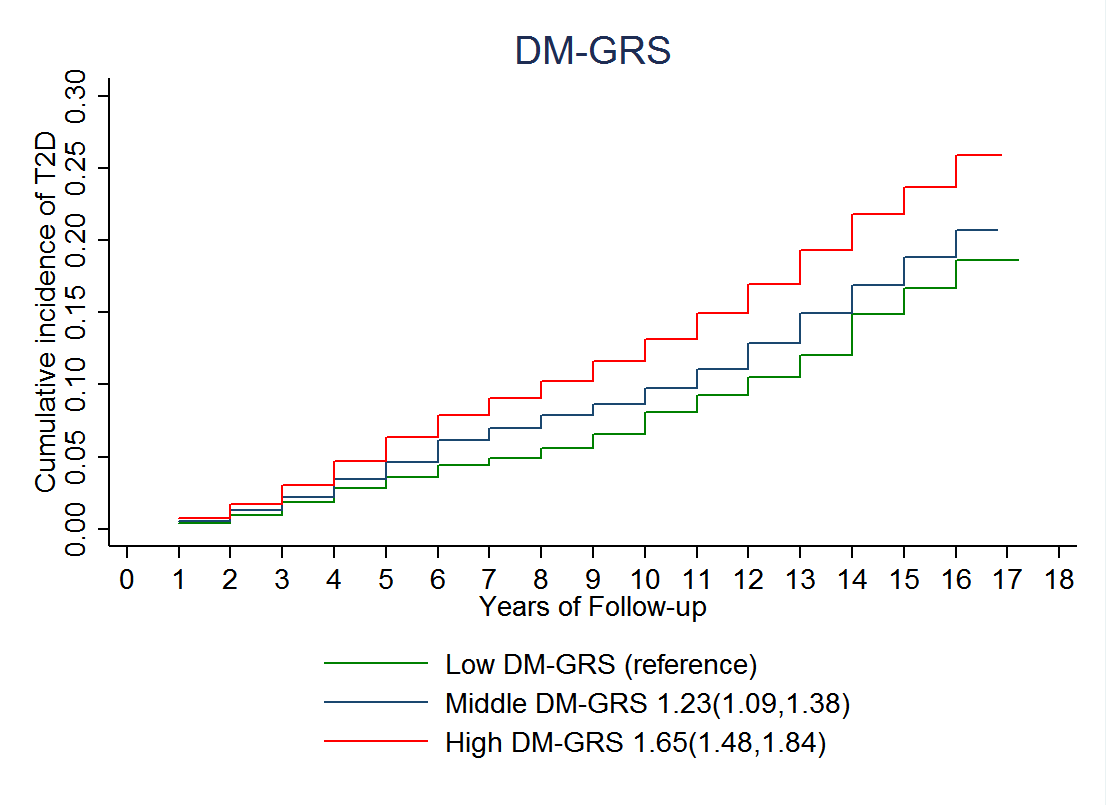


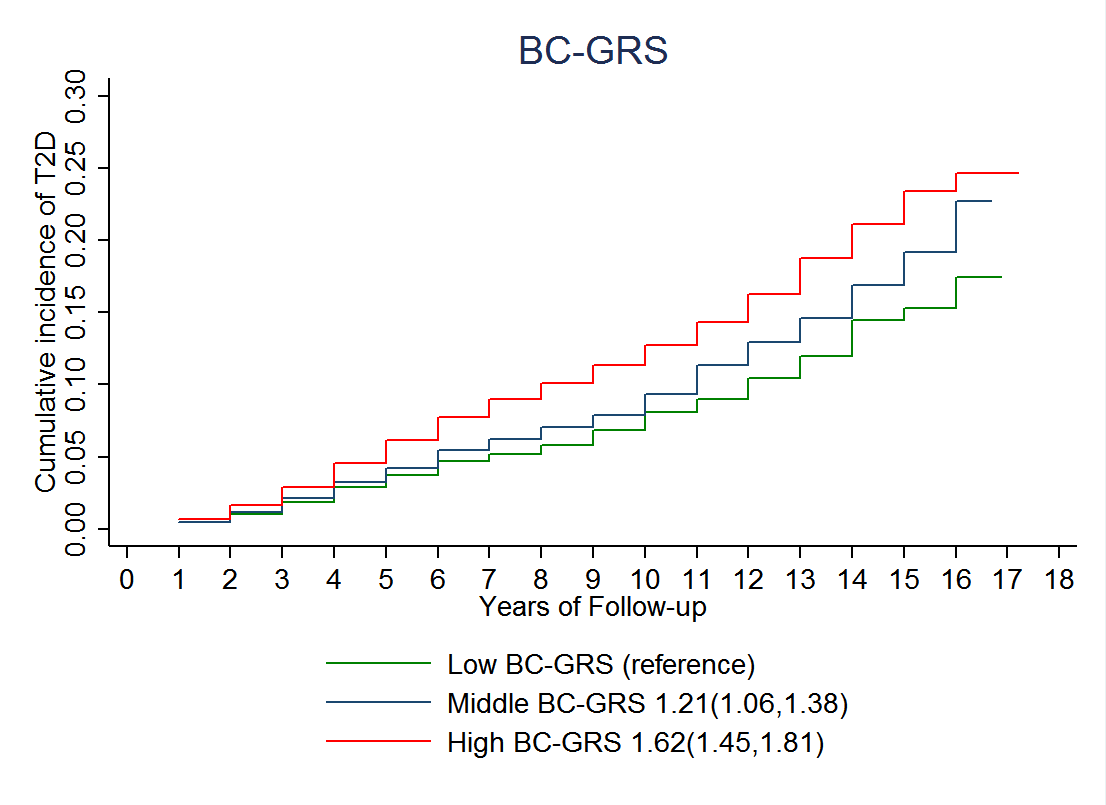

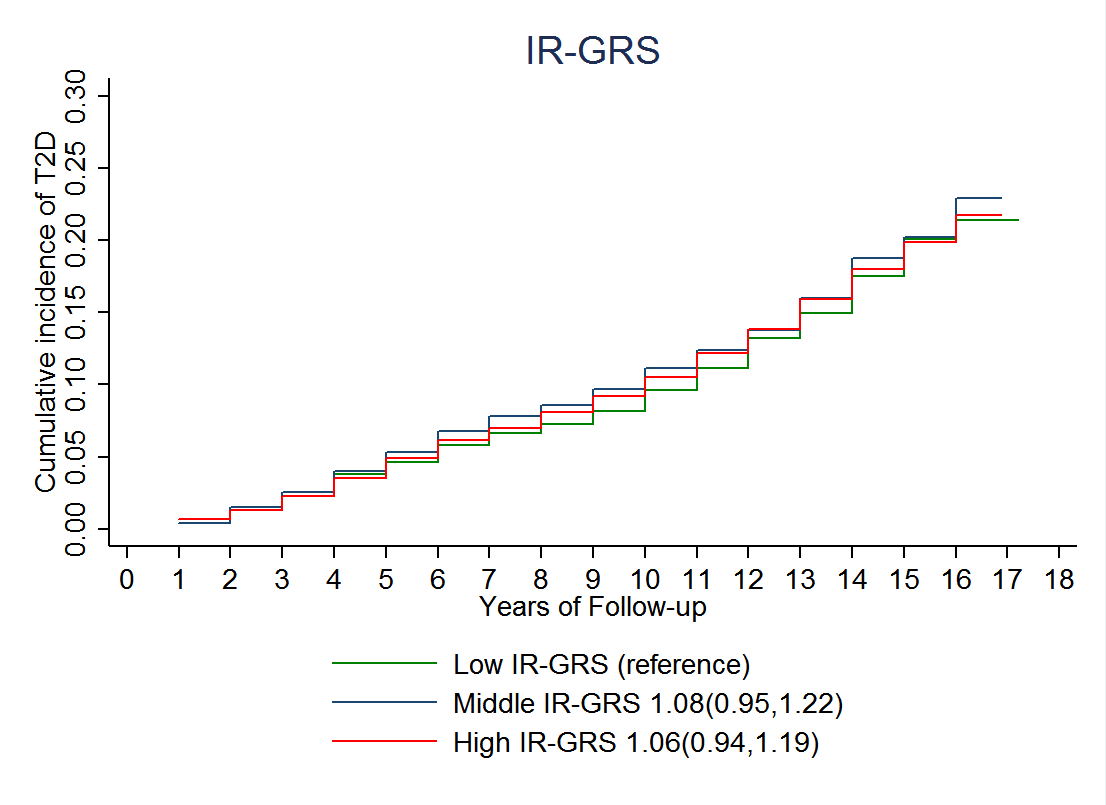


Shown are the adjusted rates of type 2 diabetes events, according to the DM-GRS, BC-GRS, IR-GRS and lifestyle score of participants. The 95% confidence intervals for the hazard ratios are provided in parentheses. Cox regression models were adjusted for sex, age, education, father dialect and years of interview, which was performed to cohort-specific population averages for each covariate. All of P for trend was <0.001, except IR-GRS (P for trend=0.47).

**Supplementary Figure 9. 10-years type 2 diabetes event rates, according to lifestyle score and DM-GRS in the CKB cohort**

Shown are adjusted 10-years cumulative incidence rates for type 2 diabetes, according to lifestyle score and DM-GRS in the pooled cohort, which were standardized to the mean of age, sex, region and data sources within study population.

**Supplementary Figure 10. 10-years type 2 diabetes event rates, according to lifestyle score and DM-GRS in the SCHS cohort**

Shown are adjusted 10-years cumulative incidence rates for type 2 diabetes, according to lifestyle score and DM-GRS in the pooled cohort, which were standardized to the mean of age, sex, region and data sources within study population.

**Supplementary Figure 11. 10-years type 2 diabetes event rates, according to lifestyle score and BC-GRS in the CKB cohort**

Shown are adjusted 10-years cumulative incidence rates for type 2 diabetes, according to lifestyle score and DM-GRS in the pooled cohort, which were standardized to the mean of age, sex, region and data sources within study population.

**Supplementary Figure 12. 10-years type 2 diabetes event rates, according to lifestyle score and IR-GRS in the CKB cohort**

Shown are adjusted 10-years cumulative incidence rates for type 2 diabetes, according to lifestyle score and DM-GRS in the pooled cohort, which were standardized to the mean of age, sex, region and data sources within study population.

**Supplementary Figure 13. 10-years type 2 diabetes event rates, according to lifestyle score and BC-GRS in the SCHS cohort**

Shown are adjusted 10-years cumulative incidence rates for type 2 diabetes, according to lifestyle score and DM-GRS in the pooled cohort, which were standardized to the mean of age, sex, region and data sources within study population.

**Supplementary Figure 14. 10-years type 2 diabetes event rates, according to lifestyle score and IR-GRS in the SCHS cohort**

Shown are adjusted 10-years cumulative incidence rates for type 2 diabetes, according to lifestyle score and DM-GRS in the pooled cohort, which were standardized to the mean of age, sex, region and data sources within study population.
